# Mitochondrial thioredoxin reductase 2 is elevated in long‐lived primate as well as rodent species and extends fly mean lifespan

**DOI:** 10.1111/acel.12596

**Published:** 2017-05-05

**Authors:** Andrew M. Pickering, Marcus Lehr, Christi M. Gendron, Scott D. Pletcher, Richard A. Miller

**Affiliations:** ^1^ Barshop Institute for Longevity and Aging Studies University of Texas Health Science Center at San Antonio San Antonio TX USA; ^2^ Department of Pathology University of Michigan Ann Arbor MI USA; ^3^ Geriatrics Center University of Michigan Ann Arbor MI USA; ^4^ Department of Molecular and Integrative Physiology University of Michigan Ann Arbor MI USA

**Keywords:** aging, *Drosophila*, lifespan, mice, mitochondria, oxidative stress, primates, rodents, thioredoxin, TXNRD2, Trxr2, TXN

## Abstract

In a survey of enzymes related to protein oxidation and cellular redox state, we found activity of the redox enzyme thioredoxin reductase (TXNRD) to be elevated in cells from long‐lived species of rodents, primates, and birds. Elevated TXNRD activity in long‐lived species reflected increases in the mitochondrial form, TXNRD2, rather than the cytosolic forms TXNRD1 and TXNRD3. Analysis of published RNA‐Seq data showed elevated TXNRD2 mRNA in multiple organs of longer‐lived primates, suggesting that the phenomenon is not limited to skin‐derived fibroblasts. Elevation of TXNRD2 activity and protein levels was also noted in liver of three different long‐lived mutant mice, and in normal male mice treated with a drug that extends lifespan in males. Overexpression of mitochondrial TXNRD2 in *Drosophila melanogaster* extended median (but not maximum) lifespan in female flies with a small lifespan extension in males; in contrast, overexpression of the cytosolic form, TXNRD1, did not produce a lifespan extension.

## Introduction

The rate at which aging leads to physiological decline, late‐life disease, and death varies greatly among species of birds, rodents, and primates. Maximum lifespan varies from 2 years to over 100 years among species of mammals. This variation is thought to represent adaptation, across evolutionary timescales, to niches that reward either rapid reproduction or slower, more sustained patterns of development and reproductive investment (Stearns, 1992). This variation in lifespan can be seen not just across the animal kingdom but within individual animal clades. Maximum lifespan among nonhuman primate species varies from 15 to 60 years. Maximum lifespan among rodent species varies from 4 to 32 years, and maximum lifespan among bird species varies from 5 to 70 years. This implies that a long lifespan has evolved multiple times in different clades. What strategies have been employed by these different groups to extend lifespan and whether these strategies are conserved or divergent among animal clades forms an interesting topic for research. Understanding the mechanisms that different species have employed to extend their lifespan has both medical implications for developing treatments to age‐associated diseases and academic interest in understanding the factors which limit species lifespan and whether such limitations are conserved across species and clades.

Comparative analysis of cultured cells from species that vary in lifespan provides a powerful tool to identify factors which may regulate the rate of aging. This approach has documented systematic variation, among species of rodents, birds, and/or primates, in proteasome structure and function (Pickering *et al*., 2015b), telomere length (Gomes *et al*., 2011), stress kinase activation (Elbourkadi *et al*., 2014), cadmium exclusion (Dostál *et al*., 2015), and resistance to oxidant injury (Harper *et al*., 2007, 2011; Pickering *et al*., 2015a). Comparisons between pairs of short‐ and long‐lived species, such as comparisons between laboratory mice and the naked mole rat (Rodriguez *et al*., 2012) or long‐ and short‐lived species of clams (Ungvari *et al*., 2011), have also been informative. In a few cases, cellular traits shown to be associated with longer lifespan in the comparative biology analyses have also been modulated by genetic, dietary, or pharmacologic interventions that extend lifespan in mice or invertebrates, including analyses of heavy metal resistance (Barsyte *et al*., 2001; Salmon *et al*., 2005; Dostál *et al*., 2015a), resistance to oxidative toxins (Salmon *et al*., 2005; Harper *et al*., 2007, 2011; Ristow & Schmeisser, 2011), and proteasome function (Rodriguez *et al*., 2012; Pickering *et al*., 2015b). The dramatic recent increase in the range of sequenced genomes, proteomes, and transcriptomes has facilitated the development of new tools to dissect the differences in cell function behind this interspecies variation. (Wright *et al*., 2010; Peng *et al*., 2015; Pickering *et al*., 2015b).

Much circumstantial evidence links cellular resistance to oxidative stress and organismal lifespan. Genetic, dietary, or drug manipulations that extend lifespan in mice, flies, and worms often increase oxidative stress resistance (Larsen, 1993; Landis *et al*., 2004; Salmon *et al*., 2005; Lithgow & Miller, 2008). Cells from longer‐lived species are often more resistant to oxidative stress than cells derived from shorter‐lived species of the same clade (Harper *et al*., 2007, 2011; Pickering *et al*., 2015a). In contrast, augmentation or knockdown of cellular antioxidant defenses seldom affects lifespan in mice (Perez *et al*., 2009). This skeptical view is consistent with the failure of a wide range of antioxidant drugs to improve health or lifespan in humans (Dolara *et al*., 2012).

Increased resistance to oxidative injury thus seems often to accompany increased longevity, but to be insufficient to increase lifespan on its own. It is possible that elevated antioxidant mechanisms may be among a suite of protective mechanisms controlled in parallel by anti‐aging drugs or mutations (Miller, 2009). A related idea proposes that some cellular processes might regulate both lifespan and stress resistance through independent mechanisms. For example, we have shown previously that cells from longer‐lived species show higher levels of proteasome function and that this increase involves increased levels of the immunoproteasome, perhaps as part of a general upregulation of the MHC class 1 antigen presentation system (Pickering *et al*., 2015b). Despite the paucity of evidence that increased antioxidant pathways can improve lifespan in rodents, there is one report of increased longevity in mice in which catalase overexpression is targeted to mitochondria, hinting that mitochondrial antioxidant defenses might be of particular importance (Schriner *et al*., 2005). Marginal lifespan effects have also been reported with overexpression of thioredoxin‐1 in mice (Mitsui *et al*., 2002; Perez *et al*., 2011).

Thioredoxin (TXN) is a small redox protein which both removes oxidants and free radicals from the cellular environment and reduces protein disulfide bonds once these are formed (Holmgren, 1989). Thioredoxin reductase (TXNRD) reduces oxidized TXN thioredoxin while simultaneously catalyzing conversion of NADPH into NADP^+^ (Holmgren, 1989). Thus, TXNRD controls the availability of reduced TXN. The TXN/TXNRD pathway also represses apoptosis through inhibition of ASK‐1 signaling (Saitoh *et al*., 1998). In mammals, there are three forms of thioredoxin reductase: cytosolic TXNRD1, mitochondrial TXNRD2, and TXNRD3, whose function is poorly defined and is thought to be predominantly expressed in the testes (Conrad *et al*., 2006). In *Drosophila*, there are two forms of thioredoxin reductase: cytosolic Trxr‐1, an orthologue of TXNRD1, and mitochondrial Trxr‐2, an orthologue of TXNRD2.

In this report, we show a correlation between TXNRD enzyme activity and species lifespan using fibroblasts from birds, rodents, and primates. In some clades, we found similar associations with glutathione reductase activity, but did not see a correlation for any of the other redox enzymes evaluated. The increase in TXNRD activity in the longer‐lived species is due to enhanced mitochondrial TXNRD2 with no change in cytosolic TXNRD1 or TXNRD3. A similar increase in TXNRD2 is seen in tissues of several models of enhanced longevity in mice, and in an analysis of mRNA levels from multiple tissues of primate species. Lastly, we demonstrate that overexpression of mitochondrial TXNRD2, but not cytosolic TXNRD1, can extend lifespan in *Drosophila melanogaster*.

These data demonstrate that augmentation of mitochondrial thioredoxin reductase 2 is a conserved approach utilized by species from a range of animal clades under selection for a long lifespan. Furthermore, we demonstrate directly that augmentation of this enzyme is able to extend organismal lifespan in *Drosophila melanogaster*. Our approach shows the power of combining comparative biology cross‐species approaches with direct interventions in model organisms as a means of discovering regulators of aging and lifespan. In addition, we identify mitochondrial Thioredoxin reductase 2 as a new target, for basic and applied research in aging.

## Results

### Thioredoxin reductase activity increases with species lifespan in cultured fibroblasts

**Figure 1:**
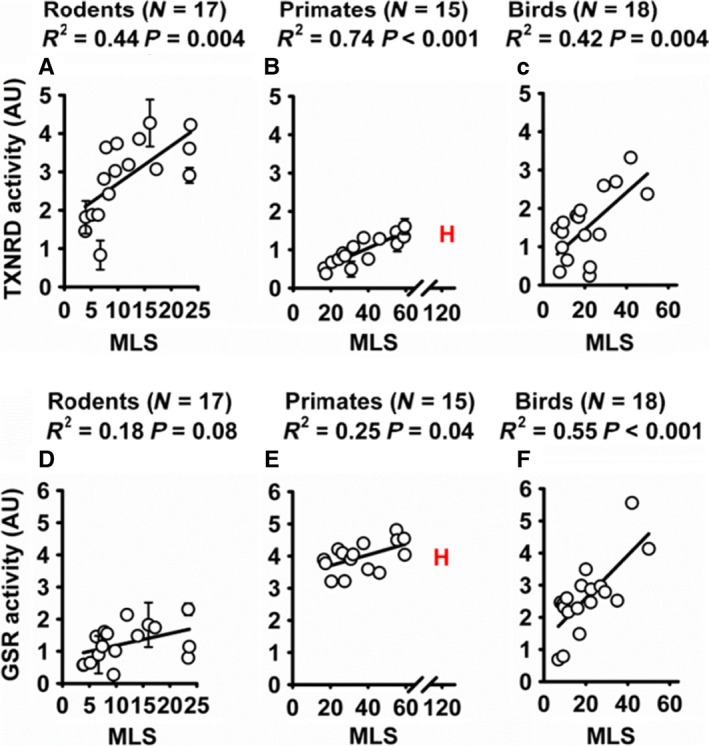
Thioredoxin reductase activity positively correlates with lifespan among rodent, primate, and bird species. Glutathione reductase activity positively correlates with lifespan among primate and bird species. (A–C) TXNRD activity in primary fibroblasts from 17 species of rodents, 15 species of primates, and 18 species of birds. (D–F) GSR activity in primary fibroblasts from 17 species of rodents, 15 species of primates, and 18 species of birds. Significance is assessed by simple linear regression analysis. Error bars if present represent multiple individuals assayed from the given species. Results for human fibroblasts are shown with an ‘H’ but are excluded from statistical analysis. MLS = maximum recorded species lifespan in years.

We were interested in whether cellular redox enzymes might be differently active in cells from long‐ and short‐lived species. To evaluate this, a collection of primary fibroblasts was developed from 17 different species of rodents, 15 species of primates, and 18 species of birds. Lysates were collected from these samples and evaluated for function of catalase, superoxide dismutase, glutathione reductase (GSR), thioredoxin reductase, glutathione peroxidase, and peroxiredoxin activity. Higher levels of total TXNRD activity were present in cells from long‐lived rodents, primates, and birds (Fig. 1A–C). A significant positive association was also seen for GSR activity among primates and bird species, with a similar trend (*P* = 0.08) for rodent species (Fig. 1D–F). We found no significant association with lifespan for catalase, superoxide dismutase, glutathione peroxidase, or peroxiredoxin activity (Fig. S1, Supporting information).

### Mitochondrial TXNRD2 and GSR are more abundant in longer‐lived species

Immunoblots were used to evaluate whether these associations between species longevity and TXNRD and GSR activity reflect an increase in protein levels of one or more forms of these enzymes. In such experiments, it is important to ensure that antibody binding affinity was not altered by interspecies sequence variation. For primates, it was possible to use antibodies for TXNRD1 and TXNRD2 that targeted epitopes conserved across all primate species for which sequence data are available. The antibodies selected for evaluation of TXNRD3 and GSR in primates recognize epitopes which varied in a small number of species, but these variations were found not to affect antibody binding affinity, based on ELISAs of the sequence variants (Fig. S2, Supporting information). For rodents, it was not possible to identify antibodies that targeted epitopes highly conserved across all of the rodent species, but we were able to use antibodies that recognized specific epitopes in subsets of the rodent species used and demonstrated that antibody binding affinity was not altered (Fig. S2, Supporting information) within each species collection. Immunoblot analysis was not performed on bird species, because the lack of sequence data precluded selection of shared target epitopes.

**Figure 2:**
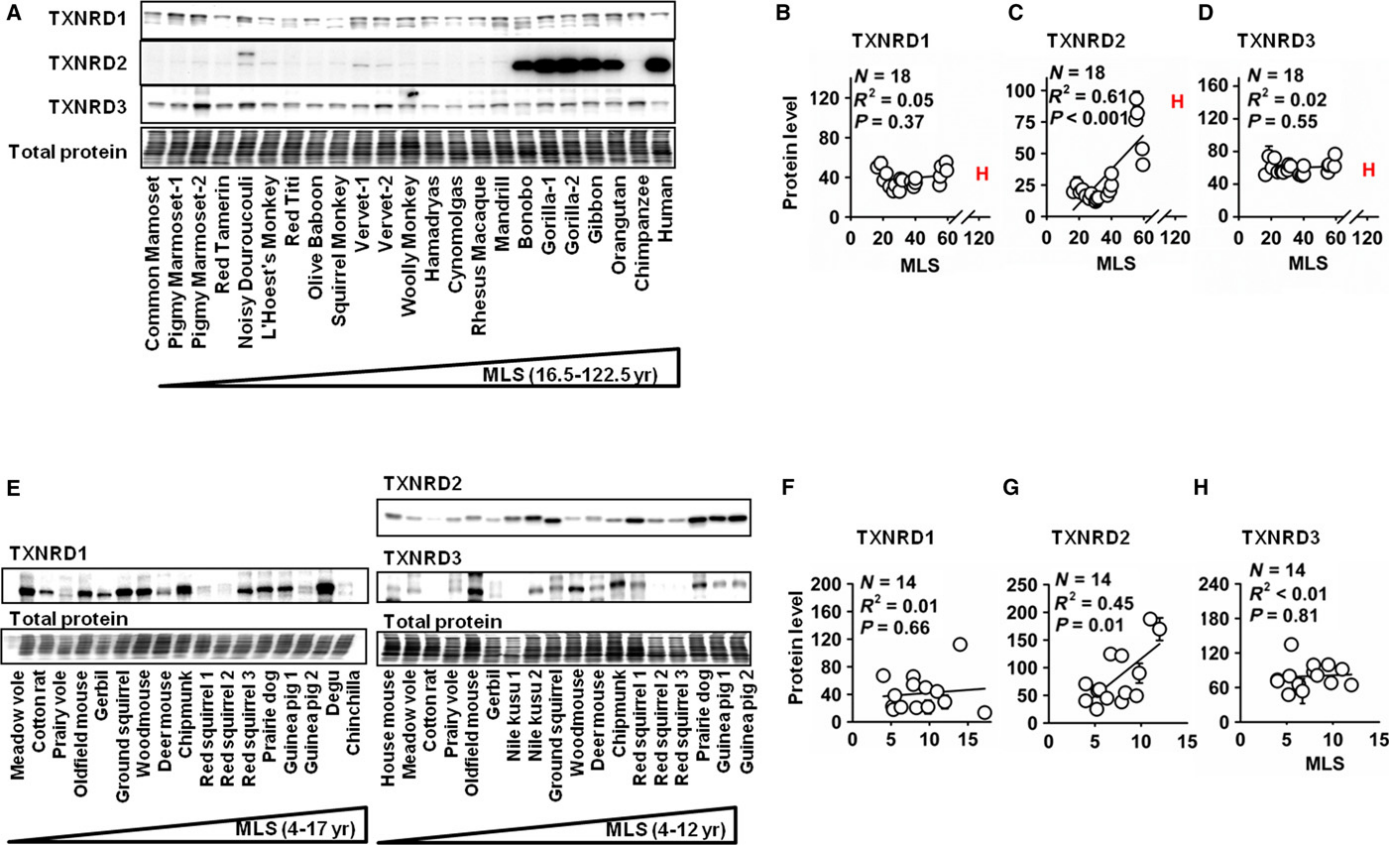
Protein levels of mitochondrial TXNRD2 have a positive correlation with lifespan among primate and rodent species. No significant lifespan association for TXNRD1 or TXNRD3. (A) Representative immunoblots for primate species. (B–D) Scatterplots of TXNRD1, TXNRD2, and TXNRD3 in primate species. (E) Representative immunoblots for rodent species. The samples were split across two blots due to antibody affinity issues; see Fig. S2 (Supporting information). (F–H) Scatterplot for TXNRD1, TXNRD2, and TXNRD3 for rodent species. Significance is assessed by simple linear regression analysis. Error bars if present represent multiple individuals assayed from the given species. Results for human fibroblasts are shown with an ‘H’ but are excluded from statistical analysis. MLS = maximum recorded species lifespan in years.

For primates, the immunoblot data showed a positive association between species lifespan and the mitochondrial isoenzyme, TXNRD2, but no association with either of the two cytosolic forms, TXNRD1 or TXNRD3 (Fig. 2A–D). Among rodents, too, only TXNRD2 showed a significant association with species longevity (Fig. 2E–H). This increase in TXNRD2 activity was associated with an increase in mitochondrial TXNRD activity in longer‐lived species with no increase in cytosolic TXNRD activity (Fig. S3, Supporting information). A parallel analysis of GSR protein levels found significant lifespan association in both rodents and primates (Fig. S4, Supporting information).

### Adjustments for phylogenetic relatedness, species body mass, and mitochondrial content

**Figure 3:**
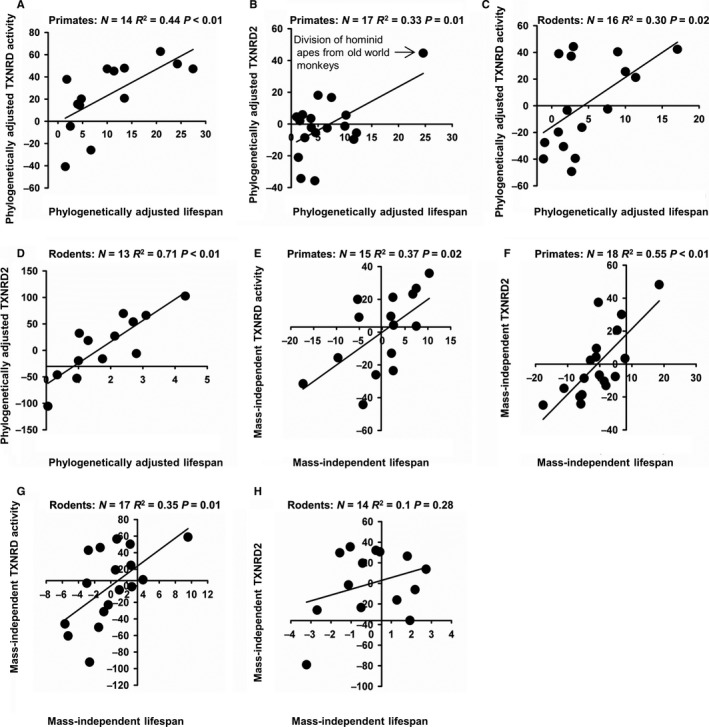
Trends mostly remain significant after correction for phylogenetic relatedness and species body mass. (A–D) Adjustment for species phylogenetic relatedness for (A) thioredoxin reductase activity in primates, (B) thioredoxin reductase 2 protein levels in primates, (C) thioredoxin reductase activity in rodents, and (D) thioredoxin reductase 2 protein levels in rodents. (E‐H) Adjustment for species body mass for (E) thioredoxin reductase activity in primates, (F) thioredoxin reductase 2 protein levels in primates, (G) thioredoxin reductase activity in rodents, and (H) thioredoxin reductase 2 protein levels in rodents.

When undertaking cross‐species evaluations, it is important to consider the effects of phylogenetic relatedness, which can lead to similar outcomes (in this case TXNRD2 levels and longevity) in species that are closely related and thus might have inherited genes that modulate both traits through a shared ancestral history, even if the outcomes are not related to one another through any causal pathway, direct or indirect. A standardized phylogenetic‐independent contrast analysis (Garland & Adolph, 1994) showed significant association for lifespan vs. TXNRD activity, and lifespan vs. TXNRD2 protein, in rodents and primates (Fig. 3A–D). The phylogenetic‐adjusted GSR regression was significant for primates (*P* = 0.02) and showed a similar but nonsignificant (*P* = 0.12) trend for rodents (Not shown). While the trends were statistically significant, it is important to note that the primate immunoblot data were heavily influenced by the diversion of the hominid apes (arrow on Fig. 3B). These apes are substantially longer‐lived and have much higher levels of TXNRD2 and GSR than other primates. No such biases were present in the other trends evaluated (Fig. 3).

Similarly, some experts have argued (Speakman, 2005) that associations between lifespan and biochemical traits can be misinterpreted if each is a reflection of interspecies differences in body mass, which is strongly associated with metabolic rates and thus with traits mechanistically tied to metabolism. Adjustments for species mass can, however, lower statistical power and increase type II (false negative) rates, because larger species of mammals and birds tend to be relatively long‐lived. Mass‐adjusted regression analyses found significant relationships between TXNRD activity, TXNRD2 protein, and GSR protein among primates, and for TXNRD activity among rodents, with parallel but not significant trends for TXNRD2 and GSR in the rodent species (Fig. 3E–H).

We also considered the possibility that the greater abundance of TXNRD2 in long‐lived species might reflect merely an increase in mitochondrial content. To test this idea, we measured levels of porin (voltage‐dependent anion channel, VDAC) as an index of mitochondrial mass and reran the regression analysis after adjustment for species differences in porin concentration. We saw no association between porin expression and species lifespan and found that the positive association between TXNRD2 protein levels and species lifespan was maintained after correction for mitochondrial content (Fig. S7, Supporting information).

### TXNRD2 mRNA is elevated in multiple tissues in long‐lived primate species

**Figure 4:**
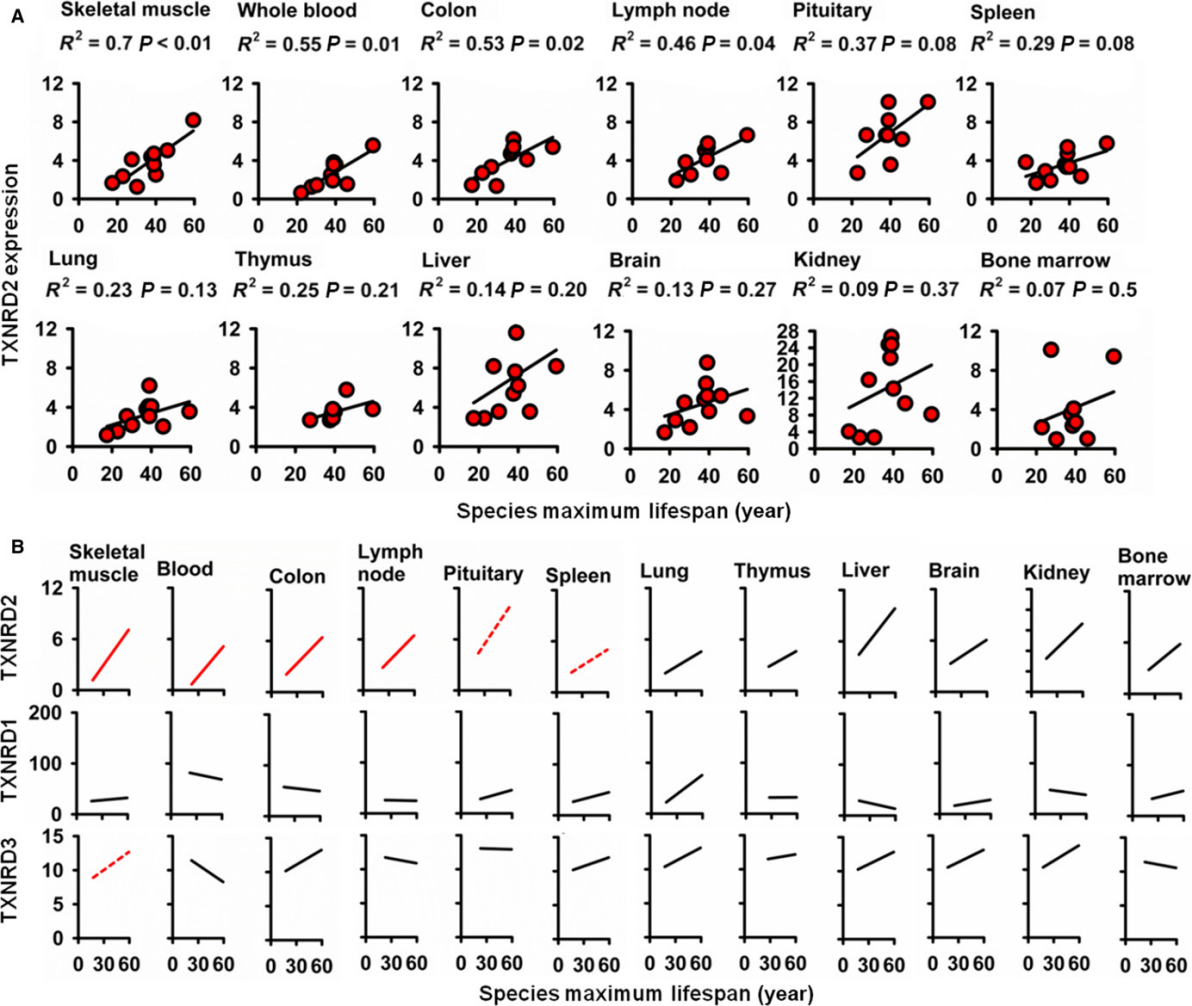
TXNRD2 mRNA (but not TXNRD1 or TXNRD3) is positively correlated with lifespan among primates in multiple tissue types. (A) Scatterplots of TXNRD2 mRNA levels plotted against maximum lifespan among primate species. mRNA levels reflect secondary analysis of RNA‐seq data from Peng *et al*. (2015). *N* varies from 6 to 12 species depending on tissue availability. (B) Summarized scatterplots of TXNRD1, TXNRD2, and TXNRD3. Red lines represent trends which are significant at *P* < 0.05, red dashed lines *P* < 0.1, and black lines *P* > 0.1. *N* varies from 6 to 12 species. Significance is based on simple linear regression analysis.

Our data in rodent and primate fibroblasts show TXNRD2 to be elevated in longer‐lived species. We wished to evaluate whether this reflected *in vivo* changes in one of more tissues in longer‐lived species. To test this, we performed a secondary analysis on a published RNA‐Seq dataset of multiple tissue types from 12 primate species (Peng *et al*., 2015). We found a positive association between species lifespan and TXNRD2 mRNA for muscle, blood, colon, lymph node, pituitary, spleen, lung, thymus, liver, brain, kidney, and bone marrow, that is, in all tissues for which RNA data were available (Fig. 4A). Despite the limited statistical power of a dataset with only 12 species, the trend reached statistical significance for muscle, blood, colon, and lymph node. No such trends were seen in the tabulated mRNA data for TXNRD1 or TXNRD2 (Fig. 4B), or for GSR (Fig. S8, Supporting information).

### Higher TXNRD activity and TXNRD2 protein levels in slow‐aging mice

**Figure 5:**
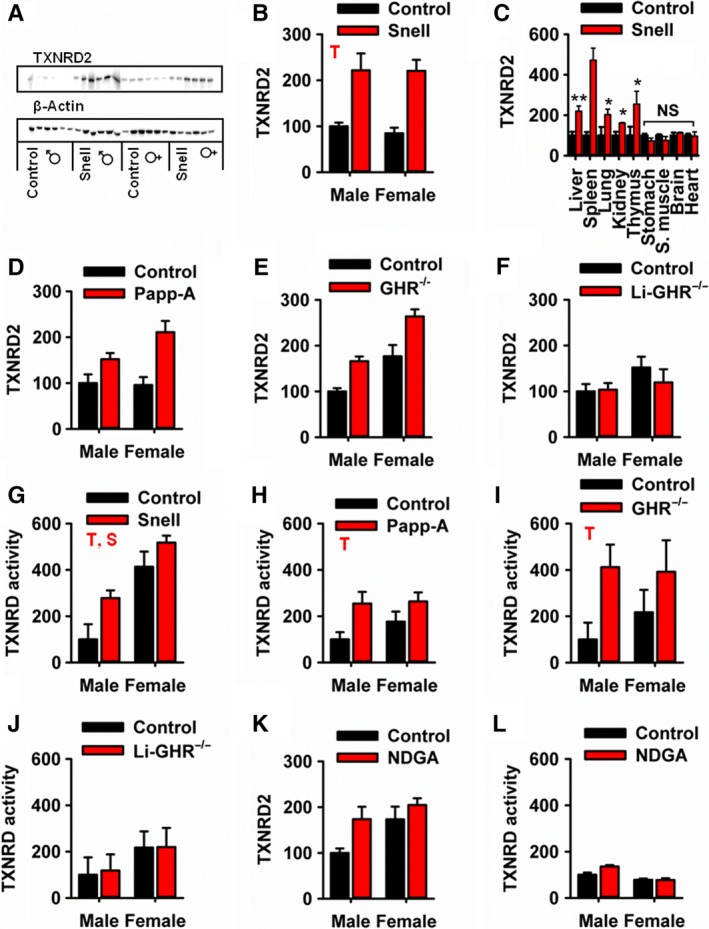
TXNRD2 protein and TXNRD activity are elevated in liver tissue from multiple long‐lived mouse models. (A) Representative immunoblots for Snell dwarf liver tissue. (B) TXNRD2 protein levels in liver tissue from Snell dwarf mice, *N* = 6. (C) TXNRD2 protein levels for multiple tissues types in male Snell dwarf mice, *N* = 3. (D–F) TXNRD2 protein levels in liver tissue from Papp‐A (*N* = 6), GHR‐/‐ (*N* = 6), and liver‐specific GHR‐/‐ (*N* = 4) mice. (G–J) TXNRD activity in liver tissue from Snell dwarf, Papp‐A, GHRKO, and liver‐specific GHR‐/‐ mice. (K–L) TXNRD2 protein levels and TXNRD activity in liver tissue from NDGA‐treated mice, *N* = 6. Significance was assessed by two‐way ANOVA. T is shown in plots where the mutation or drug has a significant effect. S is shown where sex has a significant effect. *t*‐Tests were performed only where there was a significant interaction effect. A *P*‐value table for these data is presented in Table S1 (Supporting information).

Stimulated by these comparative interspecies results, we next evaluated TXNRD in tissues of several varieties of laboratory mice in which aging had been slowed, and lifespan increased, by single gene mutations. Snell dwarf mice have a mutation in Pit‐1, which blocks development of portions of the anterior pituitary (Lewinski *et al*., 1984) and leads to lower levels of growth hormone, thyroid hormones, and IGF‐1. Snell mice live about 40% longer than littermate controls, with both sexes showing a strong effect (Flurkey *et al*., 2001). We found increased levels (~2×) of TXNRD2 in liver of male and female Snell dwarf mice (Fig. 5A,B), and significant elevation in spleen (5×), lung (2×), kidney (1.5×), and thymus (2.5×) (Fig. 5C), with no difference in stomach, muscle, brain, or heart. We also found elevations of TXNRD2 in liver of Papp‐A mice (Fig. 5D), in which deletion of a protease specific for IGF‐1 binding proteins leads to extended longevity despite normal serum levels of GH and IGF‐1. Similarly, TXNRD2 was elevated in liver tissue from growth hormone receptor knockout mice (Al‐Regaiey *et al*., 2005), in which global disruption of the growth hormone receptor leads to increased longevity in both males and females (Fig. 5E).

As a control to separate factors which are linked to the longevity phenotype in these mice from factors linked with the dwarfism phenotype, we made use of a liver‐specific growth hormone receptor knockout. These mice have very low plasma IGF‐1 and lack other hepatic responses to GH, but have a normal lifespan (Dominick *et al*., 2015). In contrast to the global GHR‐/‐ mice, the liver‐specific mutants did not show any hepatic increase in TXNRD2 (Fig. 5F), suggesting that the increase of TXNRD2 in the long‐lived mutants may depend on signals from other GH‐sensitive tissues that modulate liver gene expression.

We found that TXNRD enzyme activity parallels the results of these tests of TXNRD2 protein. We saw a significant increase in total TXNRD activity in Snell dwarf, Papp‐A, and GHR‐/‐ mice (Fig. 5G–K). Liver‐specific GHR‐/‐ had no significant change in TXNRD activity (Fig. 5K). The increases in TXNRD2 seen in Snell and GHR‐/‐ mice remained statistically significant after adjustment for mitochondrial mass using porin (Fig. S7, Supporting information), but significance was lost in Papp‐AKO mice.

In addition to the mutations which extend mouse lifespan, we also evaluated drugs that extend mouse lifespan. TXNRD2 was found to be slightly, but significantly, elevated in mice which have been treated with the drug nordihydroguaiaretic acid (NDGA), which extends mouse lifespan in males but not females (Strong *et al*., 2008; Harrison *et al*., 2014) (Fig. 5K). In these mice, a small increase in TXNRD activity was also observed in male mice treated with NDGA but not in NDGA‐treated female mice (Fig. 5L). The increase in TXNRD2 reported in NDGA‐treated male mice lost statistical significance after correction for mitochondrial load (Fig. S7, Supporting information). For this reason, we cannot discount the possibility that the change seen in NDGA‐treated mice may reflect changes in mitochondrial abundance. In addition, we evaluated mice treated with rapamycin, acarbose, and 17‐α‐estradiol (Harrison *et al*., 2014), and mice exposed to a calorie restriction diet, but found no significant change in TXNRD2 in the liver of these mice (Fig. S9, Supporting information). Another group has reported TXNRD2 (but not TXNRD1) to be elevated in skeletal muscle and heart tissue in calorically restricted rats (Rohrbach *et al*., 2006).

There is reasonable agreement between the effect size for the TXNRD2 protein levels and the TXNRD enzyme activity. Differences between these two assays are in any case not unexpected, in that the TXNRD enzyme activity reflects a mixture of TXNRD1 and TXNRD2 enzyme action in an unknown proportion. The increases in TXNRD2 seen in Snell and GHRKO mice remained statistically significant after adjustment for mitochondrial mass using porin (Fig. S7, Supporting information), but significance was lost in Papp‐AKO mice.

### Overexpression of mitochondrial Trxr‐2 (orthologue of TXNRD2) extends lifespan in *Drosophila melanogaster*

**Figure 6:**
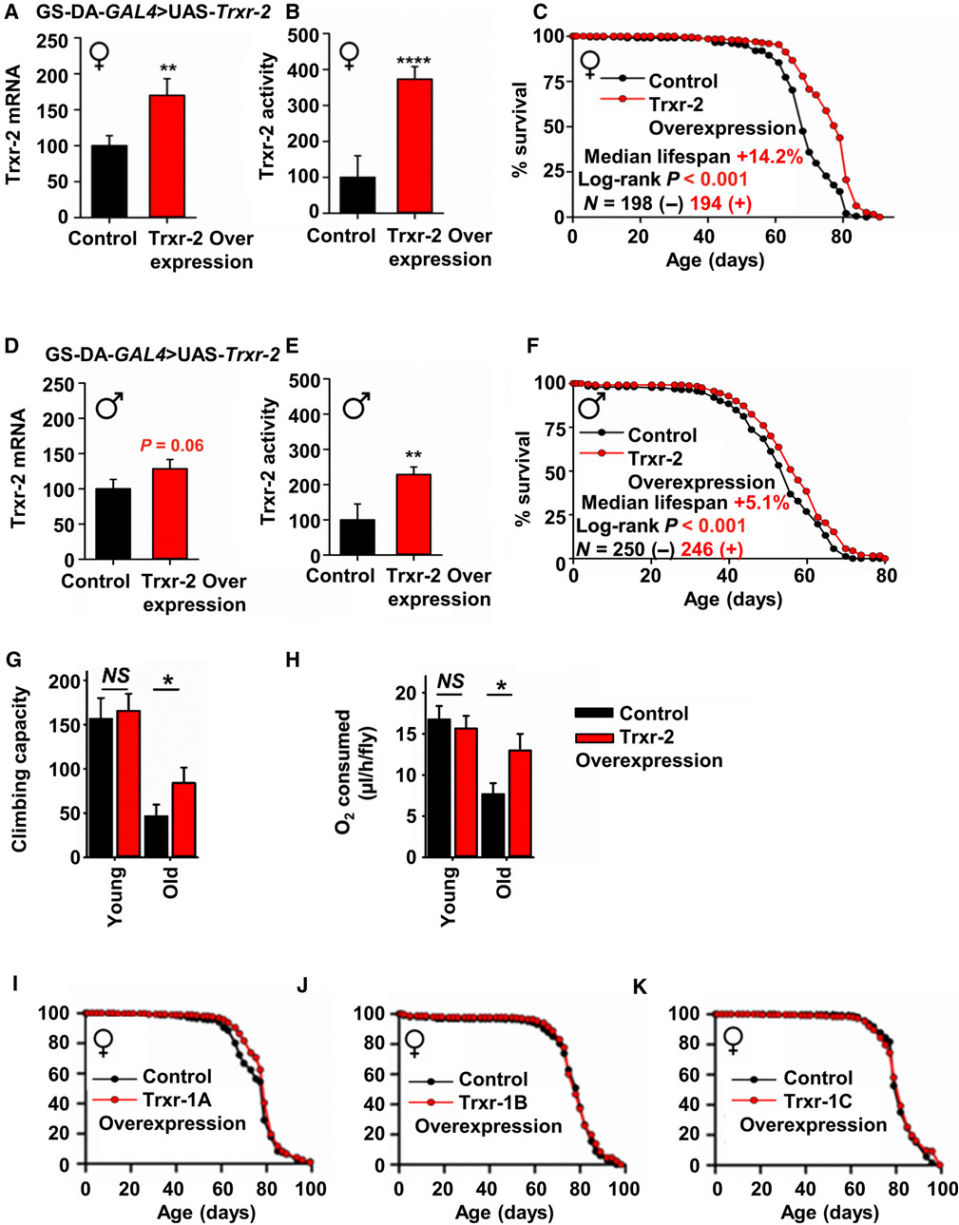
Overexpression of Trxr‐2 (orthologue of TXNRD2) increases lifespan in flies. (A) Trxr‐2 mRNA levels in GS‐Da‐*GAL4 *>** UAS‐*Trxr‐2* mated female flies in the absence and presence of RU486 inducer, *N* = 12 per condition. (B) Trxr activity in GS‐Da‐*GAL4 *>** UAS‐*Trxr‐2* mated female flies, *N* = 12 per condition. (C) Lifespan of GS‐Da‐*GAL4 *>** UAS‐*Trxr‐2* mated female flies, *N* ≈ 200. (D) Trxr‐2 mRNA levels in GS‐Da‐*GAL4 *>** UAS‐*Trxr‐2* mated male flies in the absence and presence of RU486 inducer, *N* = 12 per condition. (E) Trxr activity in GS‐Da‐*GAL4 *>** UAS‐*Trxr‐2* mated male flies, *N* = 12 per condition. (F) Lifespan of GS‐Da‐*GAL4 *>** UAS‐*Trxr‐2* mated male flies, *N* ≈ 250. (G) Number of times individually housed flies crossed a 3‐cm mark per hour measured by a TriKinetics activity monitor, *N* = 16, young = 10 days, old = 30 days. Climbing capacity was not evaluated at the older time point (60 days) because few flies are able to climb to 3 cm at this age. (H) O_2_ consumed per h, as in Yatsenko *et al*. (2014), *N* = 16, young = 10 days, old = 60 days. No significant decline in O_2_ consumption was observed at the earlier (30 days) time point. In bar charts, values are shown as mean ± SEM and significance is based on *t*‐test analysis where *P < 0.05, **P < 0.01, and ***P < 0.001, ****P < 0.0001. (I) Lifespan of GS‐Da‐*GAL4 *>** UAS‐*Trxr‐1A* mated female flies, *N* ≈ 200. (J) Lifespan of GS‐Da‐*GAL4 *>** UAS‐*Trxr‐1B* mated female flies, *N* ≈ 200. (K) Lifespan of GS‐Da‐*GAL4 *>** UAS‐*Trxr‐1C* mated female flies, *N* ≈ 200. Significance for survival data is based on the log‐rank test. UAS‐*Trxr‐2* flies are in a *yw* genetic background with a typical extinction age of 80 days. UAS‐*Trxr‐1A‐C* flies are in a *w* ^*1118*^ genetic background with a typical extinction age of 100 days.

The data above showed association between expression of TXNRD2 and lifespan across species and in several varieties of slow‐aging mice. To directly test whether increases in TXNRD2 expression are sufficient to increase lifespan, we constructed a strain of Drosophila that contained an additional copy of Trxr‐2 (*Drosophila* orthologue of TXNRD2) fused to the upstream activating sequence (UAS) for use in the bipartite GAL4‐UAS expression system. Offspring from a cross to the ubiquitous (RU486‐dependent) driver‐line GeneSwitch‐Daughterless‐*GAL4* (GS‐Da‐*GAL4*) then allowed a test for lifespan effects under adult‐specific Trxr‐2 overexpression compared to genetically identical flies from the same cohort, in which Trxr‐2 is expressed at endogenous levels (Fig. 6A,B). Overexpression of Trxr‐2 by feeding the drug RU486 was found to extend lifespan in three independent cohorts of mated female flies (Figs 6C and S9, Supporting information). To rule out off‐target effects of RU486 as the cause for lifespan differences, we evaluated flies lacking either the UAS or the GAL4 transgenic construct. We found no change in Trxr‐2 expression or activity and no lifespan extension (Fig. S10, Supporting information). RU486 treatment of GS‐Da‐*GAL4 *>* *UAS‐*Trxr‐2* flies also increased lifespan in two independent cohorts of male flies, although the effect was smaller (Figs 6D–F and S9, Supporting information), consistent with a smaller induction of Trxr‐2 in males (as shown in Fig 6A). The increase in lifespan in female flies was accompanied by diminished age‐associated reduction in muscle function and in rates of respiration (Fig. 6G,H) suggesting a potential role for TXNRD2 in age‐related physiological decline. To evaluate whether the lifespan increase was due to tissue specific effects, lifespan evaluations were also performed using the driver lines GS‐tub5‐*GAL4*, GS‐elav‐*GAL4* (neuronal‐specific), and GS‐tigs‐*GAL4* (gut‐specific), but no substantial lifespan increase was observed under these drivers (Table S2, Supporting information).

To determine whether lifespan extension is specific to the Trxr‐2/TXNRD2 isoform, we created UAS‐fused transgenes containing each of the three Drosophila Trxr‐1 (TXNRD1 orthologue) isoforms (Trxr‐1A, Trxr‐1B, and Trxr‐1C). We found that in contrast to data for Trxr‐2 overexpression, RU486‐driven overexpression of any of these isoforms failed to increase lifespan (Fig. 6I–K, Fig. S11, Supporting information). These results imply that the lifespan effect is produced specifically through overexpression of mitochondrial TXNRD2/Trxr‐2 and not the predominantly cytosolic TXNRD1/Trxr‐1. Furthermore, the failure of Trxr‐1B (which is mitochondrial) to extend lifespan might point to functional differences between Trxr‐1 and Trxr‐2 as being more pertinent to the lifespan effect than cellular distribution. Interpretation of the lifespan results is somewhat complicated by background differences, as the Trxr‐1 transgenes were only available in a w^1118^ background, and the Trxr‐2 transgene available only in a yw background. It is possible that the effects of these transgenes might differ in alternate background stocks.

## Discussion

A collection of fibroblasts from multiple species in each of several independent clades provides a powerful tool for evaluation of factors involved in evolutionary adaptation in cell traits that affect aging rates and lifespan. Early work in such systems relied on endpoints, such as stress resistance or enzymatic activity, that could be evaluated without the use of sequence‐sensitive methods (Harper *et al*., 2007, 2011; Dostál *et al*., 2015). The recent accumulation of genomic sequences from multiple species has broadened the range and depth of such comparative analysis, by allowing the use of immunoblotting for epitopes shared among species (Pickering *et al*., 2015b), and development of RNA‐seq (Peng *et al*., 2015), and proteomic technologies (Wright *et al*., 2010) for species known to have different lifespans.

In this investigation, a cross‐species survey of rodent, bird, and primate cell lines showed that levels of TXNRD correlated with species lifespan, independently in multiple clades. Further investigation revealed that the increase in activity appeared to reflect solely elevation of TXNRD2, the mitochondrial form of TXNRD, in both rodents and primate species. Analysis of a public RNA‐Seq database showed that elevated mRNA for TXNRD2 was characteristic of many internal tissues of longer‐lived primate species. Furthermore, we found higher levels of TXNRD2 in multiple tissues of long‐lived Snell dwarf mice, in two other varieties of long‐lived mutant mice, and in male mice treated with NDGA, a drug that extends lifespan only in males. The absence of TXNRD increase in liver mice with a liver‐specific GHR disruption suggested that control of hepatic TXNRD may depend upon intertissue interactions that also regulate mouse aging rate and lifespan, because liver‐specific GHR inactivation does not lead to a lifespan increase (Dominick *et al*., 2015).

Our finding of an association between TXNRD2/Trxr‐2 levels/activity and lifespan in several independent models was complemented by our finding that overexpression of Trxr‐2 in flies led to an increase in lifespan. This work presents TXNRD2/Trxr‐2 as an interesting new target for regulation of aging rates and lifespan. However, the mechanism by which TXNRD2/Trxr‐2 might increase lifespan is still in need of further investigation.

Our results are the first to suggest a role for thioredoxin reductase 2 as a regulator of lifespan in primates, mice, or flies. There have, however, been reports hinting at a role of thioredoxin reductase 1, or its substrate thioredoxin 1, in lifespan regulation. For example, Orr *et al*. (Orr *et al*., 2003) evaluated a series of transgenic flies bearing additional copies of Trxr‐1, together with catalase and/or superoxide dismutase. Lifespan in five such stocks showed small and inconsistent results, with no stock showing more than 9% increase over controls. Trxr‐1 was not tested on its own. In our study, we observed no lifespan extension under Trxr‐1 overexpression in flies. Two studies of mice have reported that overexpression of TXN‐1 (the substrate of TXNRD1) can extend mouse lifespan (Mitsui *et al*., 2002; Perez *et al*., 2011). Mitsui *et al*. reported that when a copy of human TXN‐1 fused to a β‐actin promoter is inserted into C57BL/6 mice, median lifespan is increased 35% and maximum lifespan 22%, but interpretation of this result is complicated by the very short median lifespan, 20 months, of the control stock. Perez *et al*. crossed the same allele onto the C57BL/6J stock, where the median control lifespan is 27–30 months. They found that mice hemizygous for the TXN‐1 transgene did not show a statistically significant increase in mean or median lifespan in either sex, and they saw no increase in the allele frequency in the longest‐lived 10%, although early mortality (10–25% of deaths) was significantly reduced in two cohorts of males.

Prior evidence for an important role of mitochondrial antioxidant defense as a regulator of aging/lifespan has been inconclusive (reviewed in Jang & Van Remmen, 2009). Overexpression of mitochondrial SOD2 was reported to extend lifespan in flies (Sun & Tower, 1999) and yeast (Longo *et al*., 1996). In contrast, knockdown or overexpression of mitochondrial SOD2 seems to have little effect on lifespan in mice (Jang *et al*., 2009). Overexpression of catalase specifically in mitochondria has been reported to extend mouse lifespan, while catalase targeted to other organelles did not appear to extend mouse lifespan (Schriner *et al*., 2005). However, the authors do point out that backcrossing the mitochondrial catalase line into a pure C57BL/6 background diminished the lifespan extension. Our observation of a link between mitochondrial TXNRD2 and lifespan in mammals and flies supports an argument for mitochondrial antioxidant defense as an important determinant of lifespan. However, this interpretation is complicated by our report that when mitochondrial targeted TXNRD1 (Trxr‐1B) is overexpressed in flies, no lifespan increase is observed.

TXNRD2/Trxr‐2 may affect aging and mortality through pathways other than protection from oxidant injury. For example, TXNRD2 can serve as a sensor of cellular stress and repressor of apoptosis. Its substrate, TXN‐2, represses signaling of apoptosis signal‐regulating kinase 1 (ASK‐1) (Saitoh *et al*., 1998). Here, TXN‐2 binds ASK‐1 when reduced but releases ASK‐1 when oxidized. The release of ASK‐1 causes the activation of a pro‐apoptotic response including p38 phosphorylation and JNK signaling (Tobiume *et al*., 2001). In addition, thioredoxin is a competitive inhibitor of tumor necrosis factor‐alpha (TNF‐α) signaling (Liu *et al*., 2000). TXNRD1/Trxr‐1 and TXNRD2/Trxr‐2 have distinct regulatory functions (e.g., Zhang *et al*., 2004). As a consequence, the apparent differences in the capacity to extend lifespan between TXNRD1/Trxr‐1 and TXNRD2/Trxr‐2 might reflect these functional differences instead of organelle localization.

Whether the association between lifespan and TXNRD2/Trxr‐2 is a product of improved mitochondrial oxidant defense or changes in sensitivity to apoptotic signaling will form an interesting subject for future investigation. In addition, further investigation into whether our findings in flies can be recapitulated in mice will prove highly informative.

## Experimental procedures

### Cell culture

Cells were cultured in DMEM (high‐glucose variant; Gibco‐Invitrogen, Waltham, MA, USA) supplemented with 10% heat‐inactivated fetal bovine serum and antibiotics (100 U mL^−1^ penicillin, 100 μg mL^−1^ streptomycin, and 0.25 μg mL^−1^ of amphotericin B; Gibco‐Invitrogen). Cells were incubated at 3% O_2_, hypoxic with respect to atmospheric O_2_ concentration, to mimic their normal physiological environment; the incubators were also maintained at 5% CO_2_, and 37 °C. Medium was replaced every 3–4days. For most experiments, cells were seeded at 300 000 cells mL^−1^ in either 6‐well plates or T75 flasks 48 h prior to assay. In most cases, media were replaced with serum free media 24 h prior to assay as described previously (Pickering *et al*., 2015a, 2015b). All cell lines used in experiments were primary cell fibroblast lines either developed by the University of Michigan or acquired from Coriell Cell Repository (Camden, NJ, USA) as described in more detail previously (Pickering *et al*., 2015a, 2015b).

### Thioredoxin reductase activity assay

Thioredoxin reductase activity was measured through changes in the spectral properties of thioredoxin. Thioredoxin has threefold greater fluorescence at _ex_280 nm _em_340 nm when in a reduced state in contrast to an oxidized state (Stryer *et al*., 1967). Samples were lysed in 50 mm potassium phosphate and 1 mm EDTA, pH 7.5, by freeze fractionation (coupled with sonication at 20% for 10 s on ice for Drosophila samples). 1 μg of cell lysate (based on BCA assay) was incubated with 1 μm of oxidized thioredoxin‐1 or thioredoxin‐2 (T8690, SRP6073; Sigma‐Aldrich, St. Louis, MO, USA). Fluorescence was assessed under _ex_280 nm _em_340 nm. Background fluorescence (lysate in the absence of substrate) was subtracted from readings. Measurements unless stated otherwise were of total cellular TXNRD activity.

### Fly stocks

To generate UAS‐Trxr‐1A, UAS‐Trxr‐1B, UAS‐Trxr‐1C, and UAS‐Trxr‐2 transgenic flies, cDNA constructs for the respective genes were cloned into a pUAST‐attb vector and submitted for sequencing analysis. The correctly constructed vectors were then sent to BestGene, Inc. (Chino Hills, CA, USA) for injection into attp‐containing Drosophila embryos. Flies were screened for gene overexpression through qPCR under a cross with GS‐Da‐*GAL4*. Fly stocks with robust overexpression profiles were then backcrossed into our yw genetic background for a minimum of eight generations.

### Fly survivorship assays

Fly stocks were maintained on a standard cornmeal‐based larval growth medium and maintained in a temperature‐ and humidity‐controlled environment (25 °C, 60% humidity) with a 12:12‐h light:dark cycle. Age‐matched emerged flies were collected within a 48‐h period and allowed to mate on standard media. After 48 h, the flies were lightly gassed using CO_2_ and sorted by gender (25 per vial) onto the experimental food. The experimental food consisted of either 10% sugar/yeast containing 200 μm RU‐486 (RU486+) or ethanol vehicle (RU486−). A minimum of eight independent vials were set up per gender/experimental treatment. The flies were housed in the 25 °C humidified incubator with 12:12‐h light:dark cycles for their entire lifespan. Flies were given fresh food every 2–3 days and the number of dead recorded using dLife software. This software was also used to blind and randomize the vials with respect to tray distribution.

### Study approval

All mouse studies performed were approved by the University Committee on Use and Care of Animals at the University of Michigan (Ann Arbor, Michigan, USA) on December 7, 2012.

## Funding

This research was supported by the National Institute of Aging, a division of the NIH (grants P30‐AG024824, U19‐AG023122, R01‐AG019899, R01‐AG030593, T32‐AG000114, F32‐AG049555), as well as by the Glenn Foundation for Medical Research.

## Conflict of interest

None declared.

## Supporting information


**Fig. S1** Catalase, Glutathione peroxidase, peroxiredoxinand superoxide dismutase activity is not correlative with lifespan.
**Fig. S2** TXNRD2, TXNRD2 and TXNRD3 antibody binding affinity across primate and rodent species.
**Fig. S3** Mitochondrial but not cytosolic thioredoxinreductase activity correlates with lifespan.
**Fig. S4** Protein levels of glutathione reductase has a positive correlation with lifespan amongst primate and rodent.
**Fig. S5** Raw data for mass correction in figure 3.
**Fig. S6** Raw data for phylogenetic correction in figure 3.
**Fig. S7** Correlation between thioredoxinreductase 2 protein level and lifespan remains significant after correction of mitochondrial load.
**Fig. S8** mRNA level of Glutationereductase have no consistent trends in relation to lifespan in primate tissue.
**Fig. S9** TXNRD2 protein is unchanged in liver tissue from caloric restricted, rapamycin, 17‐α‐estradiol and acarbosetreated mice.
**Fig. S10** Lifespan extension from thioredoxinreductase 2 overexpression is robust in both male and female flies.
**Fig. S11** Overexpression of Trxr‐1 (orthologue of TXNRD1) does not increase lifespan in flies.
**Table S1** *P*‐value table of two‐way ANOVA analysis for Fig. 4.
**Table S2** Table of % change in fly median & maximum lifespan as well as log‐rank significance.

## References

[acel12596-bib-0001] Al‐Regaiey KA , Masternak MM , Bonkowski M , Sun L , Bartke A . (2005) Long‐lived growth hormone receptor knockout mice: interaction of reduced insulin‐like growth factor i/insulin signaling and caloric restriction. Endocrinology 146, 851–860. 15498882 10.1210/en.2004-1120

[acel12596-bib-0002] Barsyte D , Lovejoy DA , Lithgow GJ . (2001) Longevity and heavy metal resistance in daf‐2 and age‐1 long‐lived mutants of *Caenorhabditis elegans* . FASEB J. 15, 627–634. 11259381 10.1096/fj.99-0966com

[acel12596-bib-0003] Conrad M , Bornkamm GW Brielmeier M . (2006) Mitochondrial and cytosolic thioredoxin reductase knockout mice. Selenium, Springer US: 195–206.

[acel12596-bib-0004] Dolara P , Bigagli E , Collins A . (2012) Antioxidant vitamins and mineral supplementation, life span expansion and cancer incidence: a critical commentary. Eur. J. Nutr. 51, 769–781. 22684632 10.1007/s00394-012-0389-2

[acel12596-bib-0005] Dominick G , Berryman DE , List EO , Kopchick JJ , Li X , Miller RA , Garcia GG . (2015) Regulation of mTOR activity in Snell dwarf and GH receptor gene‐disrupted mice. Endocrinology 156, 565–575. 25456069 10.1210/en.2014-1690 PMC4298324

[acel12596-bib-0006] Dostál L , Kohler WM , Penner‐Hahn JE , Miller RA , Fierke CA . (2015) Fibroblasts from long‐lived rodent species exclude cadmium. J. Gerontol. 70, 10–19. 10.1093/gerona/glu001 PMC4296162 24522391

[acel12596-bib-0007] Elbourkadi N , Austad SN , Miller RA . (2014) Fibroblasts from long‐lived species of mammals and birds show delayed, but prolonged, phosphorylation of ERK. Aging Cell 13, 283–291. 24219321 10.1111/acel.12172 PMC3954945

[acel12596-bib-0008] Flurkey K , Papaconstantinou J , Miller RA , Harrison DE . (2001) Lifespan extension and delayed immune and collagen aging in mutant mice with defects in growth hormone production. Proc. Natl Acad. Sci. USA 98, 6736–6741. 11371619 10.1073/pnas.111158898 PMC34422

[acel12596-bib-0009] Garland T , Adolph SC (1994) Why not to do 2‐species comparative‐studies – limitations on inferring adaptation. Physiol. Zool. 67, 797–828.

[acel12596-bib-0010] Gomes NM , Ryder OA , Houck ML , Charter SJ , Walker W , Forsyth NR , Austad SN , Venditti C , Pagel M , Shay JW , Wright WE . (2011) Comparative biology of mammalian telomeres: hypotheses on ancestral states and the roles of telomeres in longevity determination. Aging Cell 10, 761–768. 21518243 10.1111/j.1474-9726.2011.00718.x PMC3387546

[acel12596-bib-0011] Harper JM , Salmon AB , Leiser SF , Galecki AT , Miller RA . (2007) Skin‐derived fibroblasts from long‐lived species are resistant to some, but not all, lethal stresses and to the mitochondrial inhibitor rotenone. Aging Cell 6, 1–13. 17156084 10.1111/j.1474-9726.2006.00255.x PMC2766812

[acel12596-bib-0012] Harper JM , Wang M , Galecki AT , Ro J , Williams JB , Miller RA. (2011) Fibroblasts from long‐lived bird species are resistant to multiple forms of stress. J. Exp. Biol. 214(Pt 11), 1902–1910. 21562178 10.1242/jeb.054643 PMC3092728

[acel12596-bib-0013] Harrison DE , Strong R , Allison DB , Ames BN , Astle CM , Atamna H , Fernandez E , Flurkey K , Javors MA , Nadon NL , Nelson JF , Pletcher S , Simpkins JW , Smith D , Wilkinson JE , Miller RA . (2014) Acarbose, 17‐alpha‐estradiol, and nordihydroguaiaretic acid extend mouse lifespan preferentially in males. Aging Cell 13, 273–282. 24245565 10.1111/acel.12170 PMC3954939

[acel12596-bib-0014] Holmgren A (1989) Thioredoxin and glutaredoxin systems. J. Biol. Chem. 264, 13963–13966. 2668278

[acel12596-bib-0015] Jang YC , Van Remmen H (2009) The mitochondrial theory of aging: insight from transgenic and knockout mouse models. Exp. Gerontol. 44, 256–260. 19171187 10.1016/j.exger.2008.12.006

[acel12596-bib-0016] Jang YC , Perez VI , Song W , Lustgarten MS , Salmon AB , Mele J , Qi W , Liu Y , Liang H , Chaudhuri A , Ikeno Y , Epstein CJ , Van Remmen H , Richardson A . (2009) Overexpression of Mn superoxide dismutase does not increase life span in mice. J. Gerontol. A Biol. Sci. Med. Sci. 64, 1114–1125. 19633237 10.1093/gerona/glp100 PMC2759571

[acel12596-bib-0017] Landis GN , Abdueva D , Skvortsov D , Yang J , Rabin BE , Carrick J , Tavaré S , Tower J . (2004) Similar gene expression patterns characterize aging and oxidative stress in *Drosophila melanogaster* . Proc. Natl Acad. Sci. USA 101, 7663–7668. 15136717 10.1073/pnas.0307605101 PMC419663

[acel12596-bib-0018] Larsen PL (1993) Aging and resistance to oxidative damage in *Caenorhabditis elegans* . Proc. Natl Acad. Sci. USA 90, 8905–8909. 8415630 10.1073/pnas.90.19.8905 PMC47469

[acel12596-bib-0019] Lewinski A , Bartke A , Kovacs K , Richardson L , Smith NK . (1984) Further evidence of inactivity of hypothalamo‐pituitary‐thyroid axis in Snell dwarf mice. Anat. Rec. 210, 617–627. 6524699 10.1002/ar.1092100409

[acel12596-bib-0020] Lithgow GJ , Miller RA (2008) Determination of aging rate by coordinated resistance to multiple forms of stress The Molecular Biology of Aging. NY, USA: Cold Spring Harbor Press, 427–481.

[acel12596-bib-0021] Liu H , Nishitoh H , Ichijo H , Kyriakis JM . (2000) Activation of apoptosis signal‐regulating kinase 1 (ASK1) by tumor necrosis factor receptor‐associated factor 2 requires prior dissociation of the ASK1 inhibitor thioredoxin. Mol. Cell. Biol. 20, 2198–2208. 10688666 10.1128/mcb.20.6.2198-2208.2000 PMC110836

[acel12596-bib-0022] Longo VD , Gralla EB , Valentine JS . (1996) Superoxide dismutase activity is essential for stationary phase survival in *Saccharomyces cerevisiae*. Mitochondrial production of toxic oxygen species *in vivo* . J. Biol. Chem. 271, 12275–12280. 8647826 10.1074/jbc.271.21.12275

[acel12596-bib-0023] Miller RA (2009) Cell stress and aging: new emphasis on multiplex resistance mechanisms. J. Gerontol. A Biol. Sci. Med. Sci. 64, 179–182. 19225033 10.1093/gerona/gln072 PMC2655020

[acel12596-bib-0024] Mitsui A , Hamuro J , Nakamura H , Kondo N , Hirabayashi Y , Ishizaki‐Koizumi S , Hirakawa T , Inoue T , Yodoi J . (2002) Overexpression of human thioredoxin in transgenic mice controls oxidative stress and life span. Antioxid. Redox Signal. 4, 693–696. 12230882 10.1089/15230860260220201

[acel12596-bib-0025] Orr WC , Mockett RJ , Mockett RJ , Benes JJ , Sohal RS . (2003) Effects of overexpression of copper‐zinc and manganese superoxide dismutases, catalase, and thioredoxin reductase genes on longevity in *Drosophila melanogaster* . J. Biol. Chem. 278, 26418–26422. 12743125 10.1074/jbc.M303095200

[acel12596-bib-0026] Peng X , Thierry‐Mieg J , Thierry‐Mieg D , Nishida A , Pipes L , Bozinoski M , Thomas MJ , Kelly S , Weiss JM , Raveendran M , Muzny D , Gibbs RA , Rogers J , Schroth GP , Katze MG , Mason CE . (2015) Tissue‐specific transcriptome sequencing analysis expands the non‐human primate reference transcriptome resource (NHPRTR). Nucleic Acids Res. 43(Database issue), D737–D742. 25392405 10.1093/nar/gku1110 PMC4383927

[acel12596-bib-0027] Perez VI , Bokov A , Van Remmen H , Mele J , Ran Q , Ikeno Y , Richardson A . (2009) Is the oxidative stress theory of aging dead? Biochim. Biophys. Acta 1790, 1005–1014. 19524016 10.1016/j.bbagen.2009.06.003 PMC2789432

[acel12596-bib-0028] Perez VI , Cortez LA , Lew CM , Rodriguez M , Webb CR , Van Remmen H , Chaudhuri A , Qi W , Lee S , Bokov A , Fok W , Jones D , Richardson A , Yodoi J , Zhang Y , Tominaga K , Hubbard GB , Ikeno Y . (2011) Thioredoxin 1 overexpression extends mainly the earlier part of life span in mice. J. Gerontol. A Biol. Sci. Med. Sci. 66, 1286–1299. 21873593 10.1093/gerona/glr125 PMC3210956

[acel12596-bib-0029] Pickering AM , Lehr M , Kohler WJ , Han ML , Miller RA . (2015a) Fibroblasts from longer‐lived species of primates, rodents, bats, carnivores, and birds resist protein damage. J. Gerontol. A Biol. Sci. Med. Sci. 70, 791–799. 25070662 10.1093/gerona/glu115 PMC4481684

[acel12596-bib-0030] Pickering AM , Lehr M , Miller RA . (2015b) Lifespan of mice and primates correlates with immunoproteasome expression. J. Clin. Invest. 125, 2059–2068. 25866968 10.1172/JCI80514 PMC4463211

[acel12596-bib-0031] Ristow M , Schmeisser S (2011) Extending life span by increasing oxidative stress. Free Radic. Biol. Med. 51, 327–336. 21619928 10.1016/j.freeradbiomed.2011.05.010

[acel12596-bib-0032] Rodriguez KA , Edrey YH , Osmulski P , Gaczynska M , Buffenstein R . (2012) Altered composition of liver proteasome assemblies contributes to enhanced proteasome activity in the exceptionally long‐lived naked mole‐rat. PLoS ONE 7, e35890. 22567116 10.1371/journal.pone.0035890 PMC3342291

[acel12596-bib-0033] Rohrbach S , Gruenler S , Teschner M , Holtz J . (2006) The thioredoxin system in aging muscle: key role of mitochondrial thioredoxin reductase in the protective effects of caloric restriction? Am. J. Physiol. Regul. Integr. Comp. Physiol. 291, R927–R935. 16675629 10.1152/ajpregu.00890.2005

[acel12596-bib-0034] Saitoh M , Nishitoh H , Fujii M , Takeda K , Tobiume K , Sawada Y , Kawabata M , Miyazono K , Ichijo H . (1998) Mammalian thioredoxin is a direct inhibitor of apoptosis signal‐regulating kinase (ASK) 1. EMBO J. 17, 2596–2606. 9564042 10.1093/emboj/17.9.2596 PMC1170601

[acel12596-bib-0035] Salmon AB , Murakami S , Bartke A , Kopchick J , Yasumura K , Miller RA . (2005) Fibroblast cell lines from young adult mice of long‐lived mutant strains are resistant to multiple forms of stress. Am. J. Physiol. Endocrinol. Metab. 289, E23–E29. 15701676 10.1152/ajpendo.00575.2004

[acel12596-bib-0036] Schriner SE , Linford NJ , Martin GM , Treuting P , Ogburn CE , Emond M , Coskun PE , Ladiges W , Wolf N , Van Remmen H , Wallace DC , Rabinovitch PS . (2005) Extension of murine life span by overexpression of catalase targeted to mitochondria. Science 308, 1909–1911. 15879174 10.1126/science.1106653

[acel12596-bib-0037] Speakman JR (2005) Correlations between physiology and lifespan–two widely ignored problems with comparative studies. Aging Cell 4, 167–175. 16026331 10.1111/j.1474-9726.2005.00162.x

[acel12596-bib-0038] Stearns SC (1992) The Evolution of Life Histories. Oxford, New York: Oxford University Press.

[acel12596-bib-0039] Strong R , Miller RA , Astle CM , Floyd RA , Flurkey K , Hensley KL , Javors MA , Leeuwenburgh C , Nelson JF , Ongini E , Nadon NL , Warner HR , Harrison DE . (2008) Nordihydroguaiaretic acid and aspirin increase lifespan of genetically heterogeneous male mice. Aging Cell 7, 641–650. 18631321 10.1111/j.1474-9726.2008.00414.x PMC2695675

[acel12596-bib-0040] Stryer L , Holmgren A , Reichard P . (1967) Thioredoxin. A localized conformational change accompanying reduction of the protein to the sulfhydryl form. Biochemistry 6, 1016–1020. 4382243 10.1021/bi00856a009

[acel12596-bib-0041] Sun J , Tower J (1999) FLP recombinase‐mediated induction of Cu/Zn‐superoxide dismutase transgene expression can extend the life span of adult *Drosophila melanogaster* flies. Mol. Cell. Biol. 19, 216–228. 9858546 10.1128/mcb.19.1.216 PMC83880

[acel12596-bib-0042] Tobiume K , Matsuzawa A , Takahashi T , Nishitoh H , Morita K , Takeda K , Minowa O , Miyazono K , Noda T , Ichijo H . (2001) ASK1 is required for sustained activations of JNK/p38 MAP kinases and apoptosis. EMBO Rep. 2, 222–228. 11266364 10.1093/embo-reports/kve046 PMC1083842

[acel12596-bib-0043] Ungvari Z , Ridgway I , Philipp EE , Campbell CM , McQuary P , Chow T , Coelho M , Didier ES , Gelino S , Holmbeck MA , Kim I , Levy E , Sosnowska D , Sonntag WE , Austad SN , Csiszar A . (2011) Extreme longevity is associated with increased resistance to oxidative stress in *Arctica islandica*, the longest‐living non‐colonial animal. J. Gerontol. A Biol. Sci. Med. Sci. 66, 741–750. 21486920 10.1093/gerona/glr044 PMC3143345

[acel12596-bib-0044] Wright JC , Beynon RJ , Hubbard SJ . (2010) Cross species proteomics. Methods Mol. Biol. 604, 123–135. 20013368 10.1007/978-1-60761-444-9_9

[acel12596-bib-0045] Yatsenko AS , Marrone AK , Kucherenko MM , Shcherbata HR . (2014) Measurement of metabolic rate in *Drosophila* using respirometry. J. Vis. Exp. Jun 24;(88), e51681. 10.3791/51681 PMC4205100 24998593

[acel12596-bib-0046] Zhang R , Al‐Lamki R , Bai L , Streb JW , Miano JM , Bradley J , Min W . (2004) Thioredoxin‐2 inhibits mitochondria‐located ASK1‐mediated apoptosis in a JNK‐independent manner. Circ. Res. 94, 1483–1491. 15117824 10.1161/01.RES.0000130525.37646.a7

