# Supplementary material for: Mitochondrial thioredoxin reductase 2 is elevated in long‐lived primate as well as rodent species and extends fly mean lifespan
Source: Aging Cell. 2017 May 5;16(4):683–92. doi: 10.1111/acel.12596 (PMC5506402; doi:10.1111/acel.12596)
Supplement: Supplementary file 1 — Fig. S1 Catalase, Glutathione peroxidase, peroxiredoxinand superoxide dismutase activity is not correlative with lifespan. Fig. S2 TXNRD2, TXNRD2 and TXNRD3 antibody binding affinity across primate and rodent species. Fig. S3 Mitochondrial but not cytosolic thioredoxinreductase activity correlates with lifespan. Fig. S4 Protein levels of glutathione reductase has a positive correlation with lifespan amongst primate and rodent. Fig. S5 Raw data for mass correction in figure 3. Fig. S6 Raw data for phylogenetic correction in figure 3. Fig. S7 Correlation between thioredoxinreductase 2 protein level and lifespan remains significant after correction of mitochondrial load. Fig. S8 mRNA level of Glutationereductase have no consistent trends in relation to lifespan in primate tissue. Fig. S9 TXNRD2 protein is unchanged in liver tissue from caloric restricted, rapamycin, 17‐α‐estradiol and acarbosetreated mice. Fig. S10 Lifespan extension from thioredoxinreductase 2 overexpression is robust in both male and female flies. Fig. S11 Overexpression of Trxr‐1 (orthologue of TXNRD1) does not increase lifespan in flies. Table S1 P‐value table of two‐way ANOVA analysis for Fig. 4. Table S2 Table of % change in fly median & maximum lifespan as well as log‐rank significance. [file ACEL-16-683-s001.pdf]

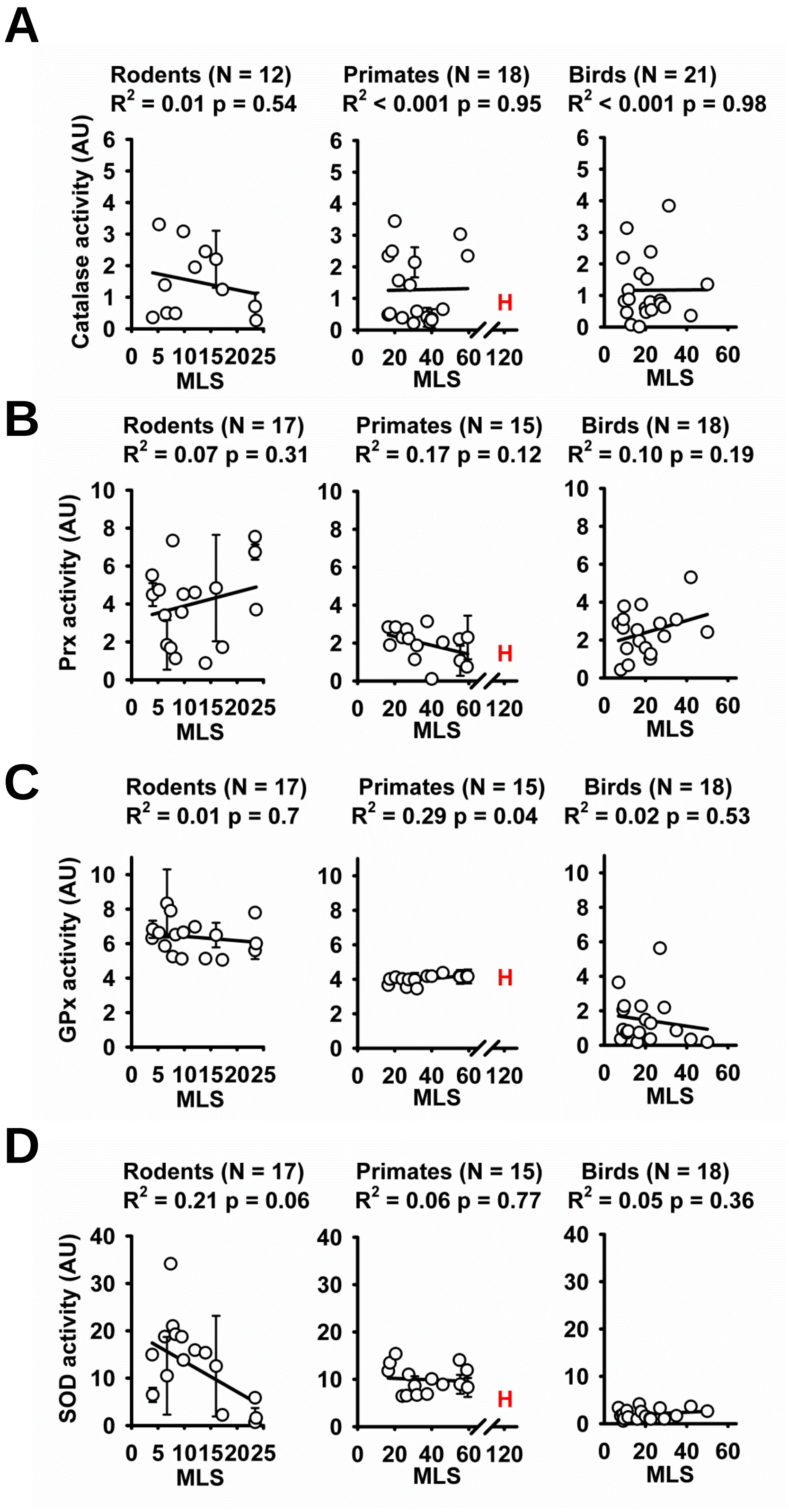

Fig. S1: Catalase, Glutathione peroxidase, peroxiredoxin and superoxide dismutase activity is not correlative with lifespan.
(A) Catalase activity in primary fibroblasts from 12 species of rodents, 18 species of primates and 21 species of birds.
(B) Peroxiredoxin activity in primary fibroblasts from 17 species of rodents, 15 species of primates and 18 species of birds.
(C) Glutatione peroxidase activity in primary fibroblasts from 17 species of rodents, 15 species of primates and 18 species of birds.
(D) Superoxide dismutase activity in primary fibroblasts from 17 species of rodents, 15 species of primates and 18 species of birds.
Significance is based on simple linear regression analysis. Error bars if present represent multiple individuals assayed in a species.
Humans are shown with an 'H' but are excluded from statistical analysis. MLS = Maximum recorded species lifespan in years

A

	Species	Family	TXNRD1	TXNRD2	TXNRD3
Human	Homo sapiens	Hominidae	TKRSGASI LQAGC	GEVTQGFALGIKCGASYAQVMRTVG IHPTCSEEVVKLRISKRSGLDPTVT	ATGERPRYLGIQGDK EYCITSDDLFSLPYC
Chimpanzee	Pan troglodytes	Hominidae	TKRSGASI LQAGC	GEVTQGFALGIKCGASYAQVMRTVG IHPTCSEEVVKLRISKRSGLDPTVT	ATGERPRYLGIQGDK EYCITSDDLFSLPYC
Bonobo	Pan paniscus	Hominidae	TKRSGASI LQAGC	GEVTQGFALGIKCGASYAQVMRTVG IHPTCSEEVVKLRISKRSGLDPTVT	ATGERPRYLGIQGDK EYCITSDDLFSLPYC
Gorilla	Gorilla gorilla	Hominidae	TKRSGASI LQAGC	No sequence data	ATGERPRYLGIQGDK EYCITSDDLFSLPYC
Orangutan	Pongo abelii	Hominidae	TKRSGASI LQAGC	GEVTQGFALGIKCGASYAQVMRTVG IHPTCSEEVVKLRISKRSGLDPTVT	ATGERPRYLGIQGDK EYCITSDDLFSLPYC
Gibbon	Nomascus leucogenys	Hylobatidae	TKRSGASI LQAGC	GEVTQGFALGIKCGASYAQVMRTVG IHPTCSEEVVKLRISKRSGLDPTVT	ATGERPRYLGIQGDK EYCITSDDLFSLHYC
Olive baboon	Papio anubis	Cercopithecidae	TKRSGASI LQAGC	GEVTQGFALGIKCGASYAQVMRTVG IHPTCSEEVVKLRISKRSGLDPTVT	ATGERPRYLGIQGDK EYCITSDDLFSLPYC
Green Monkey	Chlorocebus sabaeus	Cercopithecidae	TKRSGASI LQAGC	GEVTQGFALGIKCGASYAQVMRTVG IHPTCSEEVVKLRISKRSGLDPTVT	ATGERPRYLGIQGDK EYCITSDDLFSLPYC
Rhesus macaque	Macaca mulatta	Cercopithecidae	TKRSGASI LQAGC	GEVTQGFALGIKCGASYAQVMRTVG IHPTCSEEVVKLRISKRSGLDPTVT	ATGERPRYLGIQGDK EYCITSDDLFSLPYC
Cynomolgus	Macaca fasicularis	Cercopithecidae	TKRSGASI LQAGC	GEVTQGFALGIKCGASYAQVMRTVG IHPTCSEEVVKLRISKRSGLDPTVT	ATGERPRYLGIQGDK EYCITSDDLFSLPYC
Snub-nosed monkey	Rhinopithecus roxellana	Cercopithecidae	TKRSGASI LQAGC	GEVTQGFALGIKCGASYAQVMRTVG IHPTCSEEVVKLRISKRSGLDPTVT	ATGERPRYLGIQGDK EYCITSDDLFSLPYC
Squirrel monkey	Saimiri boliviensis	Cebidae	TKRSGASI LQAGC	GEVTQGFALGIKCGASYAQVMRTVG IHPTCSEEVVKLRISKRSGLDPTVT	ATGERPRYLGIQGDK EYCITSDDLFSLPYC
Common marmoset	Callithrix jacchus	Callitrichidae	TKRSGASI LQAGC	GEVTQGFALGIKCGASYAQVMRTVG IHPTCSEEVVKLRISKRSGLDPTVT	ATGERPRYLGIQGDK EYCITSDDLFSLPYC

B

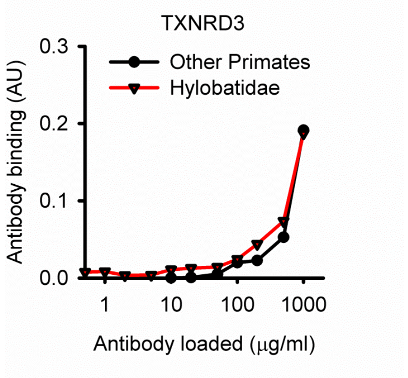

C

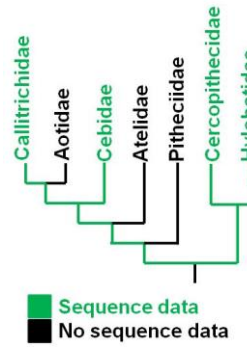

D

	Family	Subfamily	TXNRD1	TXNRD2	TXNRD3
Common Mamoset	Callitrichidae	-	√	√	√
Pigmy Marmoset	Callitrichidae	-	√	√	√
Red Tamerin	Callitrichidae	-	√	√	√
Noisy Dourooucouli	Aotidae	-	√	√	√
L'Hoest's Monkey	Cercopithecidae	Cercopithecinae	√	√	√
Red Titi	Pitheciidae	-	√	√	√
Olive Baboon	Cercopithecidae	Cercopithecinae	√	√	√
Squirrel Monkey	Cebidae	Saimiriinae	√	√	√
Vervet	Cercopithecidae	Cercopithecinae	√	√	√
Woolly Monkey	Atelidae	Atelinae	√	√	√
Hamadryas Baboon	Cercopithecidae	Cercopithecinae	√	√	√
Cynomolgas	Cercopithecidae	Cercopithecinae	√	√	√
Rhesus Macaque	Cercopithecidae	Cercopithecinae	√	√	√
Mandrill	Cercopithecidae	Cercopithecinae	√	√	√
Bonobo	Hominidae	Homininae	√	√	√
Gorilla	Hominidae	Homininae	√	√	√
Gibbon	Hylobatidae	-	√	√	√
Orangutan	Hominidae	Ponginae	√	√	√
Chimpanzee	Hominidae	Homininae	√	√	√
Human	Hominidae	Homininae	√	√	√

E

Family	Sub-family	Species	TXNRD1	TXNRD2	TXNRD3
			PHRIKATNNKGKEK	RHGQEHVEVYHAHYKPL	ATGERPRYLGIQGDKEYCITSDDLFSLPYC
Muridae		Mus musculus	PHRIVATNNKGKEK	LHGQEHVEVYHAYYKPL	ATGERPRYLGIQGDKEYCITSDDLFSLPYC
Cricetidae	Neotominae	Peromyscus maniculatus	PHKIKATNNKGKEK	LHGQEHVEVYHAYYKPL	ATGERPRYLGIQGDKEYCITSDDLFSLRYC
Cricetidae	Arvicolinae	Microtus ochrogaster	PHRIKATNNKGKEK	LHGQEHVEVYHAYYKPL	ATGERPRYLGLQGDKEYCITSDDLFSLPYC
Cricetidae	Cricetinae	Mesocricetus auratus	PHKIKATNNKGKEK	LHGQEHVEVYHAYYKPL	ATGERPQYLGIQGDKEYCITSDDLFSLPYC
Caviidae		Cavia porcellus	PHRIKATNNKGKEK	RHGQEHVEVYHAYYKPL	ATGERPRYLGIQGDKEYCITSDDLFSLPYC
Sciuridae		Ictidomys tridecemlineatus	PHRIKATNNKGKEK	RHGQEHIEVYHAYYKPL	ATGERPRYLGIPGDKEYCISSDDLFSLPYC
Chinchillidae		Chinchilla lanigera	PHRIKATNIKGKEK	RHGQENIEVYHAYYKPL	ATGERPRYLGIQGDKEYCITSDDLFSLPYC
Octodontidae		Octodon Degus	PHRIKATNNKGKEK	RHGQGNIEVYHAYYKPL	ATGERPRYLGIQGDKEYCITSDDLFSLPYC
Castoridae			NO DATA FOR FAMILY	NO DATA FOR FAMILY	NO DATA FOR FAMILY
Erethizontidae			NO DATA FOR FAMILY	NO DATA FOR FAMILY	NO DATA FOR FAMILY

F **G** **H**

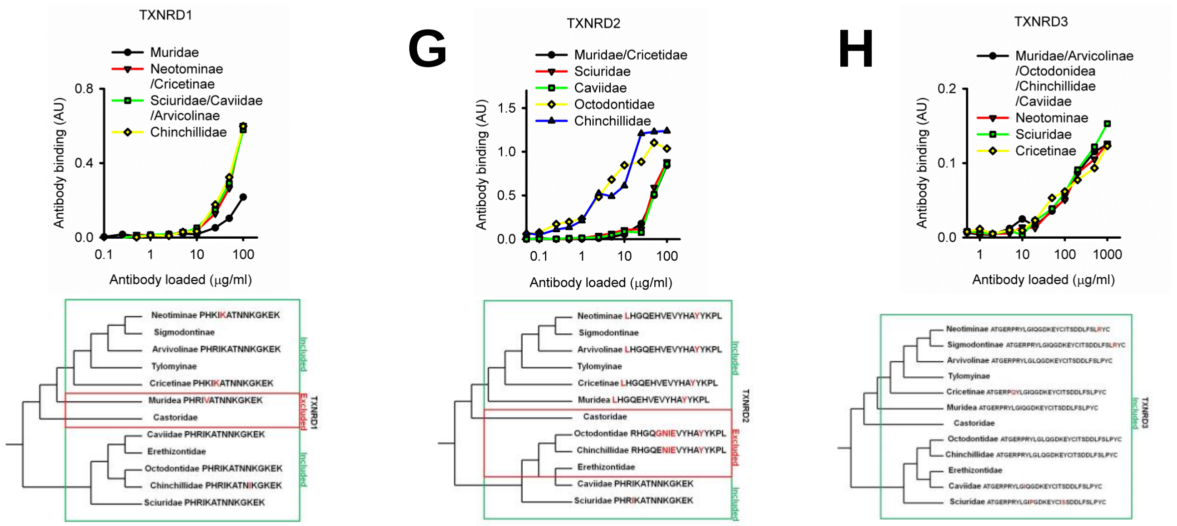

Fig. S2: TXNRD2, TXNRD2 and TXNRD3 antibody binding affinity across primate and rodent species
(A) Contrast between antibody binding epitope and published sequences on NCBI for primate species. Note Otolemur garnettii and Tarsius syrichta were not listed as all species evaluated in figure 2 were contained within the Simiiformes infraorder of which these two species are outside . **(B)** Although the TXNRD3 epitope slightly differed for Hylobatidae this did not effect antibody binding affinity. Shown through an ELISA assay with custom produced peptides. **(C)** The primate species used in figure 2 span 8 families, although sequence data is only avalable of species in 5 of these families we consider coverage sufficient to assume the sequences are conserved accross all 8 families. **(D)** List of primate species in figure 2. We considered sequence data sufficient to include all species in analysis. **(E)** Contrast between antibody binding epitope and published sequences on NCBI for rodent species. Note Blesmols (mole rats) were excluded as these considered species were considered distinct from other rodent species. **(F - H)** Differences in antibody binding affinity determined by ELISA assay against custom synthesized peptides in some species led to the exclusion of some rodent groups from analysis in figure 2.

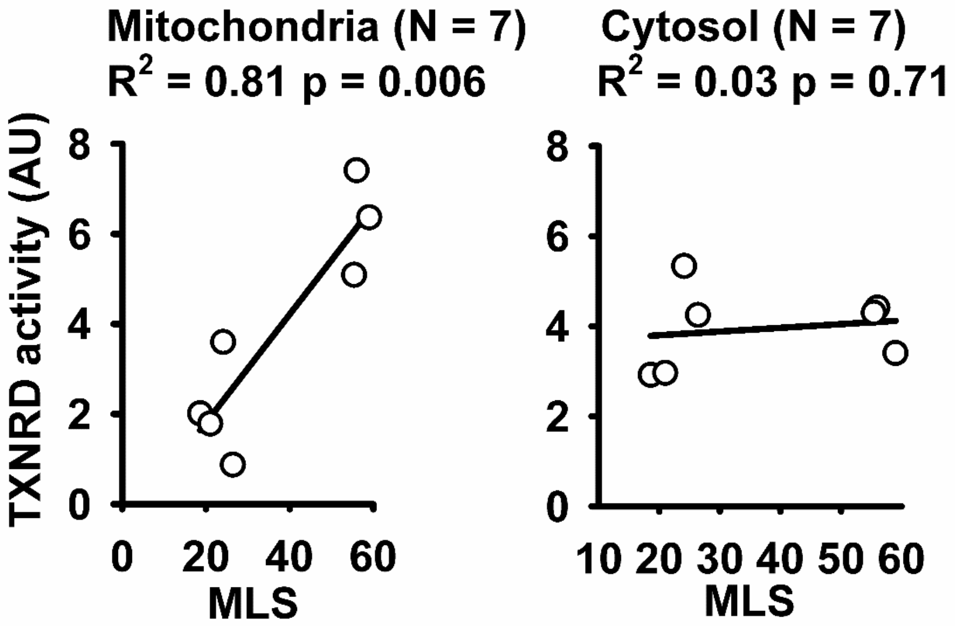

Fig. S3: Mitochondrial but not cytosolic thioredoxin reductase activity correlates with lifespan.
Thioredoxin reductase activity in primary fibroblasts from 7 species primates, Cells were divided into mitochondrial and cytosolic fractions prior to assay.

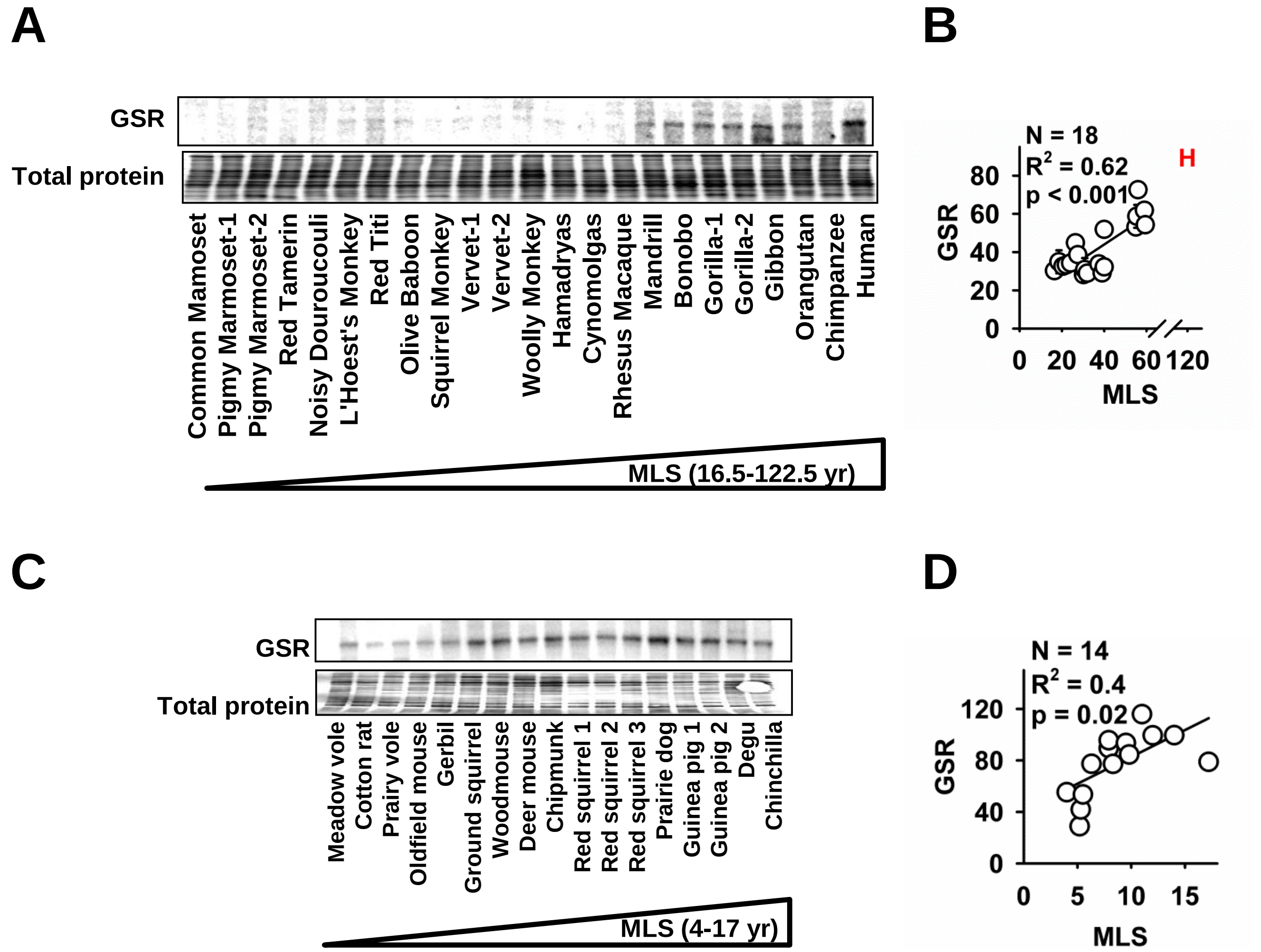

E

	Species	Family	Subfamily	GSR
Human	Homo sapiens	Hominidae	Homininae	AAGRKLAHRLFEYK EDSKLDYNNIPTVVF
Chimpanzee	Pan troglodytes	Hominidae	Homininae	AAGRKLAHRLFEYK EDSKLDYNNIPTVVF
Bonobo	Pan paniscus	Hominidae	Homininae	AAGRKLAHRLFEYK EDSKLDYNNIPTVVF
Gorilla	Gorilla gorilla	Hominidae	Homininae	AAGRKLAHRLFEYK EDSKLDYNNIPTVVF
Orangutan	Pongo abelii	Hominidae	Ponginae	AAGRKLAHRLFEYK EDSKLDYNNIPTVVF
Gibbon	Nomascus leucogenys	Hylobatidae	-	AAGRKLAHRLFEYK EDSKLDYNNIPTVVF
Snub-nosed monkey	Rhinopithecus roxellana	Cercopithecidae	Colobinae	AAGRKLAHRLFEHK EDSKLDYNNIPTVVF
Drill	Mandrillus leucophaeus	Cercopithecidae	Cercopithecinae	AAGRKLAHRLFEHK EDSKLDYNNIPTVVF
Olive baboon	Papio anubis	Cercopithecidae	Cercopithecinae	AAGRKLAHRLFEHK EDSKLDYNNIPTVVF
Grivet	Chlorocebus aethiops	Cercopithecidae	Cercopithecinae	AAGRKLAHRLFEHK EDSKLDYNNIPTVVF
Green monkey	Chlorocebus sabaeus	Cercopithecidae	Cercopithecinae	AAGRKLAHRLFEHK EDSKLDYNNIPTVVF
Crab-eating macaque	Macaca fascicularis	Cercopithecidae	Cercopithecinae	AAGRKLAHRLFEHK EDSKLDYNNIPTVVF
Rhesus macaque	Macaca mulatta	Cercopithecidae	Cercopithecinae	AAGRKLAHRLFEHK EDSKLDYNNIPTVVF
Angola colobus	Colubus angolensis	Cercopithecidae	Cercopithecinae	AAGRKLAHRLFEHK EDSKLDYNNIPTVVF
Squirrel monkey	Saimiri boliviensis	Cebidae	Saimiriinae	AAGRKLAHRLFEHK EDSKLDYNNIPTVVF
Common marmoset	Callithrix jacchus	Callitrichidae	-	AAGRKLAHRLFEHK EDSKLDYNNIPTVVF

F

			GSR
Family	Sub-family	Species	AAGRKLAHRLFECKQDSKLDYDNIPTVVF
Muridae		Mus musculus	AAGRKLAHRLFECKQDSKLDYDNIPTVVF
Cricetidae	Neotominae	Peromyscus maniculatus	AAGRKLAHRLFECKEDSKLDYDNIPTVVF
Cricetidae	Arvicolinae	Microtus ochrogaster	AAGRKLAHRLFECKKDSKLDYDNIPTVVF
Cricetidae	Cricetinae	Cricetulus griseus	AAGRKLAHRLFECKEDSKLDYDNIPTVVF
Caviidae		Cavia porcellus	AAGRKLAHRLFECKEDSKLDYDNIPTVVF
Sciuridae		Ictidomys tridecemlineatus	AAGRKLAHRLFECKEDSKLDYDNIPTVVF
Chinchillidae		Chinchilla lanigera	AAGRKLAHRLFECKEDSKLDYDNIPTVVF
Octodontidae		Octodon Degus	AAGRKLAHRLFECKEDSKLDYDNIPTVVF
Castoridae			NO DATA FOR FAMILY
Erethizontidae			NO DATA FOR FAMILY

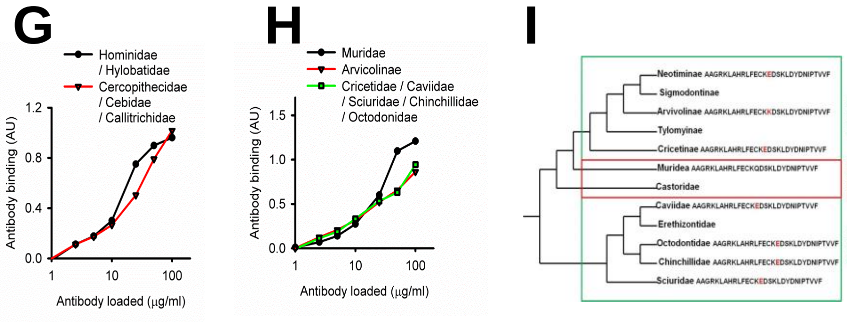

Fig. S4: Protein levels of glutathione reductase has a positive correlation with lifespan amongst primate and rodent species. (A) Representative immunoblots for primate species. **(B)** Scatterplot for GSR amongst primate species. **(C)** Representative immunoblots for rodent species. **(D)** Scatterplot for GSR amongst primate species. **(E)** Contrast between antibody binding epitope and published sequences on NCBI for primate species. **(F)** Contrast between antibody binding epitope and published sequences on NCBI for rodent species. **(G)** Slight epitope variations do not effect antibody binding affinity in primates. Shown through an ELISA assay with custom synthesized peptides **(H)** Most epitope variations do not effect antibody binding affinity in rodents though antibody binding affinity was altered in Muridea species **(I)** Muridae species and Castoridae species were excluded from evaluation to differences in antibody binding affinity in Muridea and lack of sequence information in Castoridae.

A

	maximum lifespan (yrs)	mass(g)	log_{10}(mass)	TXNRD2	mass predicted lifespan	mass predicted TXNRD2	mass independent lifespan	mass independent TXNRD2
Common Marmoset	17	255	2.41	19.56	17.56	10.10	-1.06	9.46
Pygmy Marmoset	19	124	2.09	25.43	13.17	4.86	5.43	20.57
Red Tamarin	21	501	2.70	19.26	21.66	15.00	-1.16	4.26
Noisy douroucouli	22	873	2.94	21.28	25.03	19.02	-2.93	2.26
L'Hoest's monkey	24	4700	3.67	16.42	35.25	31.24	-11.15	-14.82
Red titi	26	1120	3.05	14.10	26.54	20.83	-0.14	-6.73
Olive Baboon	28	24000	4.38	18.09	45.15	43.07	-17.65	-24.98
Common squirrel monkey	30	925	2.97	11.79	25.38	19.44	4.82	-7.66
Vervet	31	5620	3.75	13.87	36.33	32.53	-5.53	-18.66
Common woolly monkey	32	7650	3.88	14.93	38.21	34.77	-6.21	-19.84
Hamadryas Baboon	38	18000	4.26	16.70	43.40	40.98	-5.90	-24.28
Cynomolgus	39	6363	3.80	20.34	37.09	33.44	1.91	-13.10
Rhesus Macaque	40	8235	3.92	24.84	38.65	35.31	1.35	-10.47
Mandrill	40	23000	4.36	34.09	44.89	42.76	-4.89	-8.67
Bonobo	55	39925	4.60	76.85	48.24	46.76	6.76	30.09
Gorilla	55	139842	5.15	93.31	55.85	55.86	-0.45	37.46
White-handed gibbon	56	6810	3.83	82.13	37.50	33.93	18.50	48.20
Orangutan	59	64475	4.81	53.64	51.15	50.24	7.85	3.40
Chimpanzee	59	44984	4.65	41.10	48.97	47.63	10.43	-6.53

B

	maximum lifespan (yrs)	mass(g)	log_{10}(mass)	TXNRD activity	mass predicted lifespan	mass predicted TXNRD activity	mass independent lifespan	mass independent TXNRD activity
Common Marmoset	17	255	2.41	51.70	14.03	47.42	*2.47*	4.28
Coquerel's mouse lemur	17	311	2.49	37.50	15.37	50.38	*2.03*	-12.88
Red Tamarin	21	501	2.70	67.18	18.61	57.50	*1.89*	9.68
L'Hoest's monkey	24	4700	3.67	75.27	33.78	90.93	*-9.68*	-15.66
Red titi	26	1120	3.05	90.79	24.06	69.51	*2.34*	21.28
Olive Baboon	28	24000	4.38	83.77	44.84	115.28	*-17.34*	-31.51
Vervet	31	5620	3.75	49.35	35.00	93.60	*-4.20*	-44.25
Common woolly monkey	32	7650	3.88	107.30	37.09	98.20	*-5.09*	9.10
Hamadryas Baboon	38	18000	4.26	130.99	42.89	110.98	*-5.39*	20.01
Rhesus Macaque	40	8235	3.92	75.76	37.59	99.30	*2.41*	-23.54
Red-capped mangabey	46	9493	3.98	128.12	38.55	101.43	*7.45*	26.69
Bonobo	55	39925	4.60	146.13	48.29	122.88	*6.71*	23.25
Gorilla	55	139842	5.15	115.56	56.79	141.60	*-1.39*	-26.04
Orangutan	59	64475	4.81	133.72	51.54	130.04	*7.46*	3.68
Chimpanzee	59	44984	4.65	160.56	49.10	124.66	*10.30*	35.90

C

	maximum lifespan (yrs)	mass(g)	log_{10}(mass)	TXNRD2	mass predicted lifespan	mass predicted TXNRD2	mass independent lifespan	mass independent TXNRD2
mouse	4	21	1.31	70.62	5.56	40.89	-1.56	29.74
meadow vole	4	49	1.69	40.01	6.70	65.87	-2.70	-25.85
cotton rat	5	185	2.27	24.94	8.42	103.95	-3.22	-79.01
prairie vole	5	40	1.60	58.60	6.43	60.05	-1.13	-1.45
oldfield mouse	6	14	1.15	60.67	5.07	29.96	0.43	30.71
gerbil	6	53	1.73	44.78	6.80	68.22	-0.50	-23.44
nile kusu	7	110	2.04	124.52	7.75	89.05	-1.05	35.47
13-lined ground squirrel	8	173	2.24	121.66	8.33	101.98	-0.43	19.69
white footed mouse	8	23	1.36	38.13	5.71	44.19	2.19	-6.05
deer mouse	8	21	1.31	54.70	5.56	40.89	2.74	13.81
chipmunk	10	96	1.98	49.15	7.57	85.14	1.93	-35.99
red squirrel	10	200	2.30	90.08	8.52	106.18	1.28	-16.11
prairie dog	11	1125	3.05	187.77	10.76	155.70	0.24	32.07
guinea pig	12	728	2.86	169.65	10.20	143.22	1.80	26.43

D

	maximum lifespan (yrs)	mass(g)	log_{10}(mass)	TXNRD activity	mass predicted lifespan	mass predicted TXNRD activity	mass independent lifespan	mass independent TXNRD activity
Golden Hamster	4	105	2.02	72.57	9.19	133.12	-5.29	-60.55
Wild House Mouse	4	21	1.31	90.76	4.32	113.74	-0.32	-22.98
Cotton Rat	5	185	2.27	93.83	10.68	139.84	-5.68	-46.01
Gerbil	6	53	1.73	93.75	7.16	125.05	-0.86	-31.30
African Grass Rat	7	110	2.04	41.47	9.33	133.67	-2.63	-92.20
Cactus Mouse	7	25	1.40	140.83	4.91	116.09	2.49	24.73
13-lined Ground Squirrel	8	173	2.24	181.89	10.68	139.02	-2.78	42.87
Deer Mouse	8	21	1.31	121.11	4.32	113.74	3.98	7.37
Chipmunk	10	96	1.98	151.27	8.93	132.05	0.57	19.22
Red Squirrel	10	200	2.30	186.89	11.12	140.76	-1.32	46.13
Guinea Pig	12	728	2.86	159.24	14.98	156.09	-2.98	3.15
Degu	14	235	2.37	192.95	11.60	142.67	2.40	50.28
Fox Squirrel	16	800	2.90	213.74	15.26	157.21	0.74	56.53
Chinchilla	17	643	2.81	153.51	14.60	154.61	2.60	-1.10
American Beaver	23	20250	4.31	145.42	24.90	195.54	-1.50	-50.12
North American Porcupin	23	8600	3.93	180.48	22.35	185.38	1.05	-4.91
Gray squirrel	24	533	2.73	211.28	14.04	152.39	9.56	58.89

Fig. S5 Raw data for mass correction in figure 3. Raw and mass corrected data for: **(A)** TXNRD2 protein content amongst species of primates, **(B)** TXNRD2 protein content amongst species of rodent, **(C)** TXNRD activity amongst species of primates, **(D)** TXNRD activity amongst species of rodent. 'Predicted' values are based on the linear regression of Log10(species body mass) plotted against species maximum lifespan, TXNRD activity and TXNRD2 protein levels. "mass-independent' values are the predicted values subtracted from the actual values (residuals from the above plots).

A

	MLS	TXNRD activity
Marmoset	16.5	51.7
Coquerel's mouse lemur	17.4	37.5
Red tamarin	20.5	67.18
L'Hoest's monkey	24.1	75.27
Red titi	26.4	90.79
Olive baboon	27.5	83.77
Vervet	30.8	49.35
Woolly monkey	32	107.3
Hamadryas baboon	37.5	130.99
Rhesus macaque	40	75.76
Red-capped mangabey	46	128.12
Bonobo	55	146.13
Gorilla	55.4	115.56
Orangutan	59	133.72
Chimpanzee	59.4	160.56

		Predicted traits of ancestor			Phylogenetic contrast	
Node	Species connected by node	MYA	MLS	TXNRD activity	MLS Contrast	TXNRD Contrast
A	Marmoset to Tamerin	16	18.5	59.4	4.0	15.5
B	Node A to Woolly monkey	23	21.7	70.6	13.5	47.9
C	Node B to Red titi	26	22.1	72.7	4.8	20.2
D	L'Hoest's monkey to Vervet	7	27.5	62.3	6.7	-25.9
E	Olive baboon to Hamadryas baboon	1	32.5	107.4	10.0	47.2
F	Node E to Red-capped mangabey	5	38.5	116.6	13.5	20.7
G	Node F to Rhesus macaque	7	38.8	107.5	1.5	-40.8
H	Node D to Node G	10	33.1	84.9	11.4	45.2
I	Bonobo to Chimpanzee	3	57.2	153.3	4.4	14.4
J	Node I to Gorilla	9	56.5	138.2	1.8	37.8
K	Node J to Orangutan	21	57.4	136.6	2.5	-4.5
L	Node H to Node K	31	49.6	119.9	24.3	51.7
M	Node C to Node L	43	38.2	100.4	27.4	47.2
N	Node M to Mouse lemur	78	31.8	80.9	20.8	62.9

B

	MLS	TXNRD activity
American beaver	23.4	145.42
Wild house mouse	4	90.76
African grass rat	6.7	41.465
Gerbil	6.3	93.745
Cactus mouse	7.4	140.825
Deer mouse	8.3	121.105
Cotton rat	5.2	93.825
Golden hamster	3.9	72.565
Ground squirrel	7.9	181.885
Chipmunk	9.5	151.27
Red squirrel	9.8	186.89
Fox squirrel	16	213.74
Gray squirrel	23.6	211.28
Guinea pig	12	159.235
Porcupine	23.4	180.475
Chinchilla	17.2	153.51
Degu	14	192.95

		Predicted traits of ancestor			Phylogenetic contrast	
Node	Species connected by node	MYA	MLS	TXNRD activity	MLS Contrast	TXNRD Contrast
A	House mouse to African grass rat	11	5.4	66.1	2.7	-49.3
B	Node A to Gerbil	18	5.6	73.8	-1.0	-27.6
C	Cactus mouse to Deer mouse	7	7.9	131.0	0.9	-19.7
D	Node C to Cotton rat	18	6.8	116.9	2.7	37.1
E	Node D to Golden hamster	20	6.6	112.8	2.9	44.3
F	Node B to Node E	21	6.3	103.1	1.0	39.0
G	Node F to American beaver	65	13.2	120.2	17.1	42.3
H	Guinea pig to Porcupine	37	17.7	169.9	11.4	21.2
I	Chinchilla to Degu	34	15.6	173.2	3.2	-39.4
J	Node H to Node I	38	17.3	170.5	2.1	-3.4
K	Fox squirrel to Grey squirrel	8	19.8	212.5	7.6	-2.5
L	Node K to Red squirrel	11	17.7	207.0	10.0	25.6
M	Ground squirrel to Chipmunk	11	8.7	166.6	1.6	-30.6
N	Node L to Node M	46	13.2	186.8	9.0	40.4
O	Node J to Node N	67	14.9	180.0	4.1	-16.3
P	Node G to Node O	69	14.3	160.0	-1.1	-39.9

C

	MLS	TXNRD2
Common Mamoset	16.5	19.56
Pigmy Marmoset	18.6	25.43
Red Tamerin	20.5	19.26
Noisy Douroucouli	22.1	21.28
L'Hoest's Monkey	24.1	16.42
Red Titi	26.4	14.10
Olive Baboon	27.5	18.09
Squirrel Monkey	30.2	11.79
Vervet	30.8	13.87
Woolly Monkey	32	14.93
Hamadryas Baboon	37.5	16.70
Cynomolgas	39	20.34
Mandrill	40	34.09
Rhesus Macaque	40	24.84
Bonobo	55	76.85
Gorilla	55.4	93.31
Gibbon	56	82.13
Orangutan	59	53.64
Chimpanzee	59.4	41.10

		Predicted traits of ancestor			Phylogenetic contrast	
Node	Species connected by node	MYA	MLS	TXNRD2	MLS Contrast	TXNRD2
A	Pigmy Marmoset to Common	5	17.6	22.5	2.1	5.9
B	Red Tamerin to Noisy Douroucouli	14	21.3	20.3	1.6	2.0
C	Rhesus Macaque to Cynomolgas	4	39.5	22.6	1.0	4.5
D	Node A to Node B	16	20.7	20.6	3.8	-2.2
E	Node D to Squirrel monkey	20	22.9	18.6	9.5	-8.8
F	Node E to Woolly monkey	23	23.9	19.0	9.1	-3.7
G	Red titi to Node F	26	24.1	18.5	2.5	-4.9
H	Olive baboon to Hamadryas baboon	1	32.5	17.4	10.0	-1.4
I	Node H to Mandrill	5	36.0	25.2	7.5	16.7
J	Node C to Node I	7	37.8	23.9	3.5	-2.6
K	Vervet to L'hoesti monkey	7	27.5	15.1	6.7	-2.5
L	Node J to Node K	10	32.1	19.1	10.3	8.8
M	Chimpanzee to Bonobo	3	57.2	59.0	4.4	-35.8
N	Node M to Gorilla	9	56.5	72.8	1.8	-34.3
O	Node N to Orangutan	18	57.3	66.2	2.5	-19.2
P	Node O to Gibbon	21	57.2	68.2	1.3	-16.0
Q	Node L to Node P	31	49.5	53.1	25.1	49.1
R	Node G to Node Q	43	38.8	38.5	25.4	34.6

D

	MLS	TXNRD2
House mouse	4	70.62
Nile kusu	6.7	124.52
Gerbil	6.3	44.78
Oldfield mouse	5.5	60.67
Deer mouse	8.3	54.70
White-footed mouse	7.9	38.13
Cotton rat	5.2	24.94
Meadow vole	4	40.01
Prairie vole	5.3	58.60
13-lined ground squirrel	7.9	121.66
Prairie dog	11	187.77
Chipmunk	9.5	49.15
Red squirrel	9.8	90.08
Guinea pig	12	169.65

		Predicted traits of ancestor			Phylogenetic contrast	
Node	Species connected by node	MYA	MLS	TXNRD2	MLS Contrast	TXNRD2
A	Degu to Chinchilla	34	15.6	133.8	3.2	-31.1
B	Node A to Guinea pig	38	15.3	137.0	3.6	-15.4
C	Oldfield mouse to Deer mouse	1	6.9	98.1	2.8	35.5
D	Node C to White-footed mouse	4	7.3	117.5	1.0	45.3
E	Node D to Cotton rat	18	2.8	27.4	2.1	74.2
F	Meadow vole to Prairie vole	1	4.7	72.9	1.3	-20.1
G	Node E to Node F	19	3.0	31.2	1.9	45.5
H	Node G to Gerbil	21	3.1	34.0	3.3	84.8
I	Ground squirrel to Prairie dog	9	9.5	154.1	3.1	39.5
J	Node I to Chipmunk	11	9.5	152.0	0.1	-13.9
K	Node J to Red squirrel	46	9.6	141.1	0.3	-25.1
L	Node B to Node K	67	12.0	139.4	5.7	-4.1
M	Node H to Node L	69	11.6	134.5	8.9	105.4

Fig. S6: Raw data for phylogenetic correction in figure 3. Raw and phylogenetically contrasted data for: **(A)** TXNRD activity amongst species of primates, **(B)** TXNRD activity amongst species of rodent, **(C)** TXNRD2 protein content amongst species of primates, **(D)** TXNRD2 protein content amongst species of rodent. A phylogeny was constructed for the species used in each data set (weighted based on millions of years, MYA, divergence). Values for common ancestors at each node were calculated. Phylogenetic contrasts were performed between each species and it's closest relative. All contrasts involved the subtraction of the shorter lived species from the longer lived species to reduce bias to the distribution.

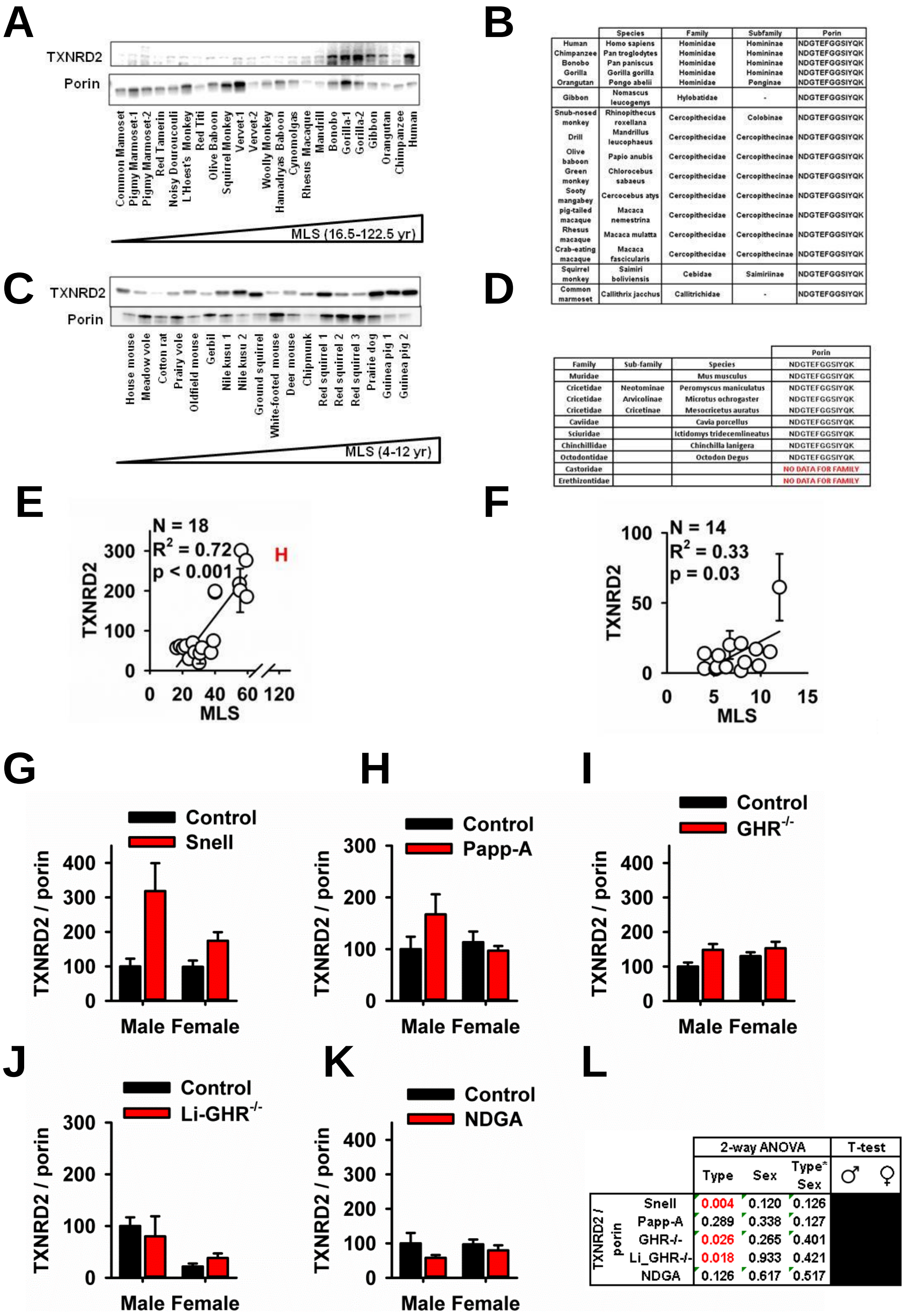

	Species	Family	Subfamily	Porin
Human	Homo sapiens	Hominidae	Homininae	NDGTEFGGSIYQK
Chimpanzee	Pan troglodytes	Hominidae	Homininae	NDGTEFGGSIYQK
Bonobo	Pan paniscus	Hominidae	Homininae	NDGTEFGGSIYQK
Gorilla	Gorilla gorilla	Hominidae	Homininae	NDGTEFGGSIYQK
Orangutan	Pongo abelii	Hominidae	Ponginae	NDGTEFGGSIYQK
Gibbon	Nomascus leucogenys	Hylobatidae	-	NDGTEFGGSIYQK
Snub-nosed monkey	Rhinopithecus roxellana	Cercopithecidae	Colobinae	NDGTEFGGSIYQK
Drill	Mandrillus leucophaeus	Cercopithecidae	Cercopithecinae	NDGTEFGGSIYQK
Olive baboon	Papio anubis	Cercopithecidae	Cercopithecinae	NDGTEFGGSIYQK
Green monkey	Chlorocebus sabaeus	Cercopithecidae	Cercopithecinae	NDGTEFGGSIYQK
Sooty mangabey	Cercocebus atys	Cercopithecidae	Cercopithecinae	NDGTEFGGSIYQK
pig-tailed macaque	Macaca nemestrina	Cercopithecidae	Cercopithecinae	NDGTEFGGSIYQK
Rhesus macaque	Macaca mulatta	Cercopithecidae	Cercopithecinae	NDGTEFGGSIYQK
Crab-eating macaque	Macaca fascicularis	Cercopithecidae	Cercopithecinae	NDGTEFGGSIYQK
Squirrel monkey	Saimiri boliviensis	Cebidae	Saimiriinae	NDGTEFGGSIYQK
Common marmoset	Callithrix jacchus	Callitrichidae	-	NDGTEFGGSIYQK

			Porin
Family	Sub-family	Species	NDGTEFGGSIYQK
Muridae		Mus musculus	NDGTEFGGSIYQK
Cricetidae	Neotominae	Peromyscus maniculatus	NDGTEFGGSIYQK
Cricetidae	Arvicolinae	Microtus ochrogaster	NDGTEFGGSIYQK
Cricetidae	Cricetinae	Mesocricetus auratus	NDGTEFGGSIYQK
Caviidae		Cavia porcellus	NDGTEFGGSIYQK
Sciuridae		Ictidomys tridecemlineatus	NDGTEFGGSIYQK
Chinchillidae		Chinchilla lanigera	NDGTEFGGSIYQK
Octodontidae		Octodon Degus	NDGTEFGGSIYQK
Castoridae			NO DATA FOR FAMILY
Erethizontidae			NO DATA FOR FAMILY

TXNRD2 / porin	2-way ANOVA: Type	Sex	Type* Sex	T-test: ♂	♀
Snell	0.004	0.120	0.126		
Papp-A	0.289	0.338	0.127		
GHR-/-	0.026	0.265	0.401		
Li_GHR-/-	0.018	0.933	0.421		
NDGA	0.126	0.617	0.517		

Fig. S7: Correlation between thioredoxin reductase 2 protein level and lifespan remains significant after correction of mitochondrial load. (A) Representative immunoblots for primate species. **(B)** Contrast between porin antibody binding epitope and published sequences on NCBI for primate species. **(C)** Representative immunoblots for rodent species. **(D)** Contrast between porin antibody binding epitope and published sequences on NCBI for rodent species. **(E)** Scatterplot TXNRD2 for primates normalized to porin **(F)** Scatterplot TXNRD2 for rodents normalized to porin. **(G – K)** Thioredoxin reductase 2 protein levels in liver tissue from Snell dwarf, Papp-A, GHR-/-, liver-specific GHR-/- and NDGA treated mice normalized to porin. (L) p-value table of two-way ANOVA analysis for B-M). T-test were run only when a significant interaction term was observed. Values are shown in red when $p < 0.05$.

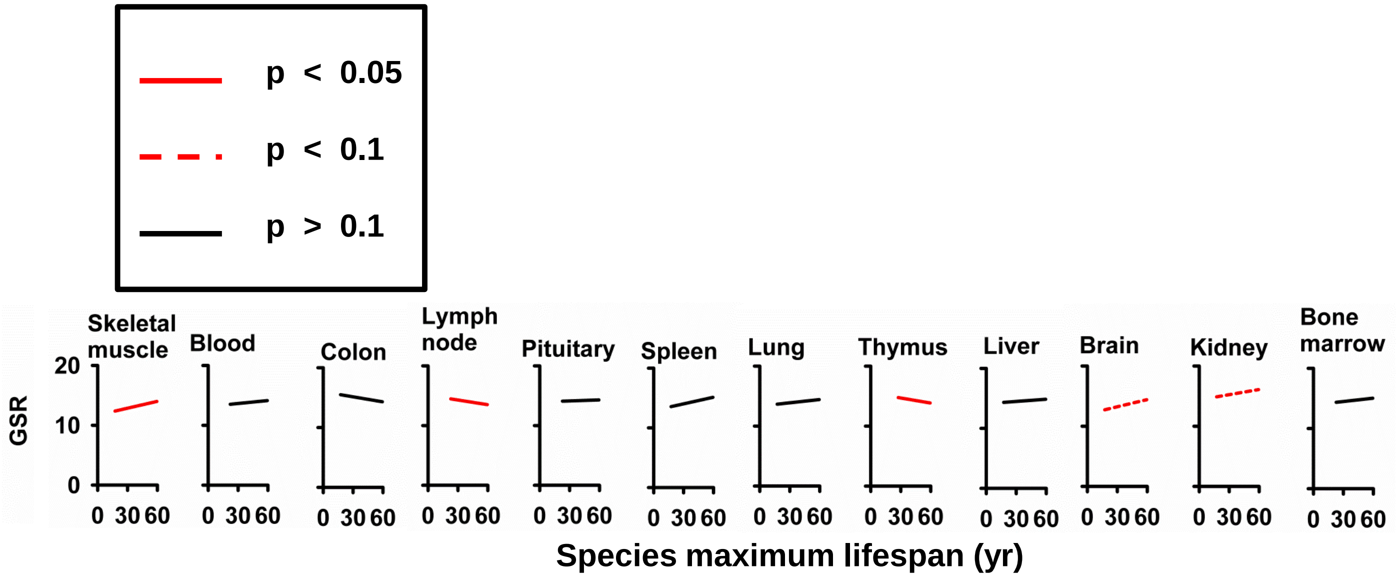

Fig. S8: mRNA level of Glutatione reductase have no consistent trends in relation to lifespan in primate tissue. Summarized scatterplots of GSR. Red lines represent trends which are significant at $p < 0.05$, red dashed lines $p < 0.1$, black lines $p > 0.1$. N varies from 6 – 12 species. Significance is based on simple linear regression analysis. Plots represents a secondary analysis of RNAseq data from (Peng et al., 2015). N varies from 6 – 12 species depending on tissue availability.

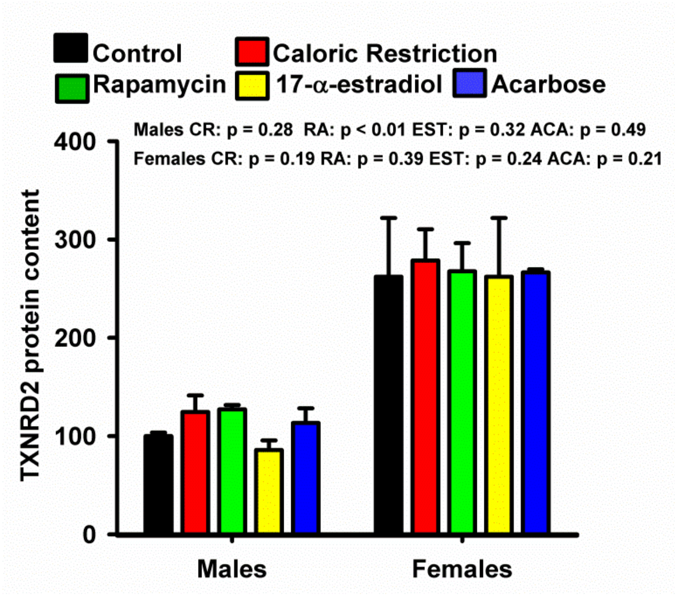

Fig. S9: TXNRD2 protein is unchanged in liver tissue from caloric restricted, rapamycin, 17-α-estradiol and acarbose treated mice. TXNRD2 protein levels in liver tissue from 12 month old mice treated with the above interventions (initiated at 4 months of age) N = 4.

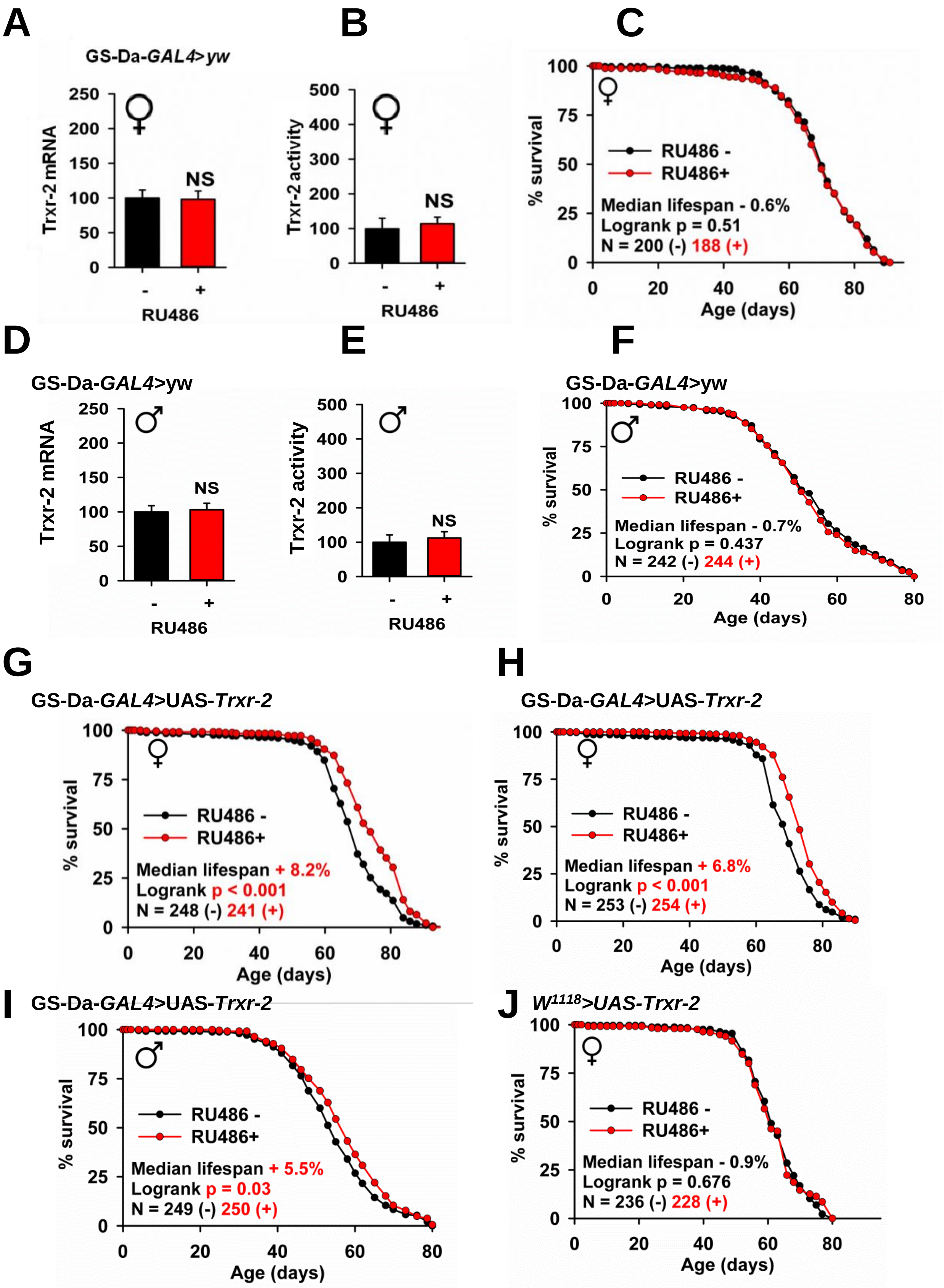

Fig. S10 Lifespan extension from thioredoxin reductase 2 overexpression is robust in both male and female flies.
(A) Trxr-2 mRNA levels in GS-Da-*GAL4>yw* mated female flies in the absence and presence of the RU486 which serves to induce the transgene. N = 12 per condition. **(B)** Thioredoxin reductase activity in GS-Da-*GAL4>yw* mated *f*emale flies. N = 12 per condition. **(C)** Lifespan of GS-Da-*GAL4>yw mated* male flies, N ≈ 200. **(D)** Trxr-2 mRNA levels GS-Da-*GAL4>yw* mated male flies. Flies are of the same genetic background as those in A - C minus the transgene this serves as a control for off target effects. N = 12 per condition. **(E)** Thioredoxin reductase activity in GS-Da-*GAL4>yw mated* male flies. N = 12 per condition. **(F)** Lifespan of GS-Da-*GAL4>yw* mated male flies, N ≈ 250. **(G-H)** Two independent repeats of the lifespan of GS-Da-*GAL4*>UAS-*Trxr-2 mated fe*male flies. **(I)** Independent repeats of the lifespan of GS-Da-*GAL4*> UAS-*Trxr-2* mated male flies. (J) Lifespan of w^{1118}>UAS-*Trxr-2* mated female flies, N ≈ 250 (control for off target effects).

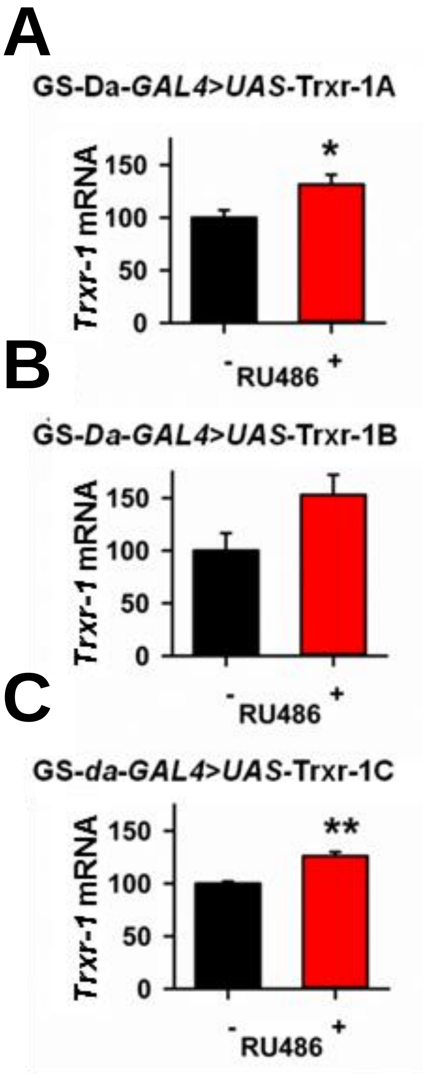

Fig. S11: Overexpression of Trxr-1 (orthologue of TXNRD1) does not increase lifespan in flies.
(A) Trxr-1 mRNA levels in GS-Da-*GAL4*>UAS-*Trxr-1A* mated female flies in the absence and presence of the RU486 inducer. N = 3 per condition. **(B)** Trxr-1 mRNA levels in GS-Da-*GAL4*>UAS-*Trxr-1B* mated female flies in the absence and presence of the RU486 inducer. N = 3 per condition. **(C)** Trxr-1 mRNA levels in GS-Da-*GAL4*>UAS-*Trxr-1C* mated female flies in the absence and presence of the RU486 inducer. N = 3 per condition.

		2-way ANOVA			T-test	
		Type	Sex	Type* Sex	♂	♀
TXNRD2	Snell	< 0.001	0.723	0.761		
	Papp-A	< 0.001	0.051	0.342		
	GHR-/-	< 0.001	< 0.001	0.510		
	Li_GHR-/-	0.450	< 0.001	0.453		
	NDGA	0.008	0.018	0.433		
TXNRD activity	Snell	< 0.001	< 0.001	0.012	0.037	0.065
	Papp-A	0.009	0.311	0.413		
	GHR-/-	0.011	0.217	0.022	0.036	0.131
	Li_GHR-/-	0.9	0.192	0.922		
	NDGA	0.069	0.001	0.035	0.022	0.311

Table S1. p-value table of two-way ANOVA analysis for figure 4. T-tests were run only when a significant interaction term was observed. Values are shown in red when $p < 0.05$.

		GS-Da-*GAL4*		GS-Tub5-*GAL4*		GS-elav-GAL4		GS-tigs-*GAL4*	
		Median (50%)	Maximum (90%)	Median (50%)	Maximum (90%)	Median (50%)	Maximum (90%)	Median (50%)	Maximum (90%)
**UAS-*Trxr-2***	Median (% change)	+ 9.7	+ 5.8	- 0.5	+ 3.7	+ 3.7	+ 0.7	+ 2.6	+ 2.3
		(average of 3 lifespans)							
	Logrank (p-value)	< 0.001		0.04		0.02		< 0.001	
**UAS-*Trxr-1A***	Median (% change)	+ 0.9	+ 0.2	+ 0.2	+ 0.1				
	Logrank (p-value)	0.03		0.265					
**UAS-*Trxr-1B***	Median (% change)	- 1.1	+ 2.3	+ 2.9	+ 5.3	- 0.5	+ 0.4	+ 0.5	0.0
	Logrank (p-value)	0.53		< 0.001		0.937		0.241	
**UAS-*Trxr-1C***	Median (% change)	+ 1.0	+ 2.6	- 3.0	- 0.2				
	Logrank (p-value)	0.331		0.01					
yw	Median (% change)	- 0.6	+ 0.3	- 3.4	- 0.3	+ 1.0	+ 2.1	- 2.0	0.0
	Logrank (p-value)	0.51		0.84		0.124		0.265	

Table S2: Table of % change in fly median & maximum lifespan as well as log-rank significance. Median lifespan is defined as the age at which 50% of flies have died. Maximum lifespan is defined as the age at which 90% of flies have died.
